# DNA Sensor ABCF1 Phase Separates With cccDNA to Inhibit Hepatitis B Virus Replication

**DOI:** 10.1002/advs.202409485

**Published:** 2024-11-05

**Authors:** Caiyue Ren, Zhaoying Zhang, Yutong Dou, Yang Sun, Zhendong Fu, Liyuan Wang, Kai Wang, Chengjiang Gao, Yuchen Fan, Shuguo Sun, Xuetian Yue, Chunyang Li, Lifen Gao, Xiaohong Liang, Chunhong Ma, Zhuanchang Wu

**Affiliations:** ^1^ Key Laboratory for Experimental Teratology of Ministry of Education and Department of Immunology School of Basic Medical Sciences Cheeloo Medical College Shandong University Jinan Shandong 250012 China; ^2^ Department of Hepatology Qilu Hospital Cheeloo Medical College Shandong University Jinan Shandong 250012 China; ^3^ Department of Human Anatomy, Histology and Embryology School of Basic Medicine Tongji Medical College Huazhong University of Science and Technology Wuhan Hubei 430030 China; ^4^ Department of Cellular Biology School of Basic Medical Sciences Shandong University Jinan Shandong 250012 China; ^5^ Key Laboratory for Experimental Teratology of the Ministry of Education Department of Histology and Embryology School of Basic Medical Sciences Shandong University Jinan Shandong 250012 China

**Keywords:** ABCF1, cccDNA, HBV, HBx, phase separation, transcriptional machinery

## Abstract

Hepatitis B virus (HBV) covalently closed circular DNA (cccDNA) contributes to viral persistence and recurrence, however, how the host innate immune system responds to cccDNA is still less known. Here, based on cccDNA‐hepatic proteins interaction profiling, DNA sensor ATP‐binding cassette subfamily F member 1 (ABCF1) is identified as a novel cccDNA‐binding protein and host restriction factor for HBV replication. Mechanistically, ABCF1 recognizes cccDNA by KKx4 motif and forms phase‐separated condensates by the poly‐glutamine (PolyQ) region of the N‐terminal intrinsically disordered low‐complexity domain (LCD). Subsequently, ABCF1‐cccDNA phase separation not only activates the type I/III interferon (IFN‐I/III) pathway but also prevents Pol II accumulation on cccDNA to inhibit HBV transcription. In turn, to sustain viral replication, HBV reduces ABCF1 expression by HBx‐mediated ubiquitination and degradation of SRY‐box transcription factor 4（SOX4）, leading to defects in SOX4‐mediated upregulation of ABCF1 transcription. Taken together, the study shows that ABCF1 interacts with cccDNA to form phase separation that dually drives innate immune signaling and HBV transcriptional inhibition. These findings shed new light on the understanding of host defense against cccDNA and provide a novel promising therapeutic strategy for HBV infection.

## Graphical abstract

This study identifies ABCF1 as a novel HBV restriction factor. Mechanistically, ABCF1 recognizes cccDNA to trigger phase separation via its LCD domain, thereby dually activating IFN response and insulating the loading of Pol II on cccDNA. In turn, HBx represses ABCF1 expression to facilitate viral replication. These findings shed new light on the understanding of host defense against HBV cccDNA.

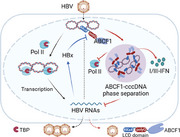

## 1 Introduction

Chronic hepatitis B virus (HBV) infection is a major cause of liver cirrhosis and hepatocellular carcinoma (HCC), which account for more than 880 000 deaths annually worldwide.^[^ ^1^ ^]^ Covalently closed circular DNA (cccDNA) is the sole transcription template driving HBV replication and plays a vital role in viral persistence and recurrence after discontinued treatment.^[^ ^2^ ^]^ Thus, clearance of intranuclear cccDNA is the ultimate goal of the HBV cure. However, cccDNA exists as a stable minichromosome and resists available antiviral drugs, making it more difficult to achieve the ultimate goal of the HBV cure.^[^ ^2^ ^]^ Recent clinical data estimate that cccDNA has a relatively short turnover of several months,^[^ ^3^ ^]^ offering the possibility of achieving HBV cure through the exhaustion of the cccDNA pool combined with strategies to repress HBV replication. Therefore, it is essential to explore novel host factors which contribute to cccDNA sensing and HBV replication repression.

It has been accepted that innate immune activation can effectively inhibit HBV replication and eliminate cccDNA. However, HBV is considered as a “stealth” hepatotropic virus that does not invoke strong IFN‐I/III responses^[^ ^4^ ^]^ and bypasses innate immune recognition by multiple pathways.^[^ ^5^ ^]^ Cytoplasmic pattern recognition receptors (PRRs), such as retinoic acid‐inducible gene I (RIG‐I) and melanoma differentiation‐associated protein 5 (MDA5), sense HBV RNAs and activate IFN‐III responses to suppress HBV replication.^[^ ^6^, ^7^ ^]^ HBV cccDNA can be sensed by interferon‐inducible protein 16 (IFI16) to induce IFN production.^[^ ^8^ ^]^ Otherwise, except for innate immune recognition, PRRs also function as direct antiviral factors. RIG‐I counteracts the interaction of HBV P protein with pgRNA to suppress viral replication^[^ ^6^ ^]^ and IFI16 promotes the epigenetic suppression of HBV cccDNA.^[^ ^8^ ^]^ However, RIG‐I, IFI16, and other PRRs have been reported to be inhibited by HBV.^[^ ^5^, ^8^, ^9^, ^10^ ^]^ IFN‐α is the only licensed drug that can induce a prolonged inhibition of cccDNA transcription. However, only 20–40% of chronic hepatitis B (CHB) patients achieve HBeAg seroconversion after 48 weeks of IFN‐α treatment.^[^ ^11^ ^]^ Thus, it would be beneficial to identify DNA sensors that recognize cccDNA and contribute to HBV inhibition in ways beyond IFN‐α.

The liquid–liquid phase separation (LLPS) has emerged as a fundamental principle to organize cellular biological processes and plays an important role in innate immunity and viral life cycles.^[^ ^12^ ^]^ The replication of DNA viruses, such as herpesvirus, papillomavirus, or adenovirus, occurs in intranuclear inclusion bodies (IBs), which are membrane‐free spherical structural formed by LLPS. Viral DNA binding to cGAS induces the formation of liquid‐like condensates that promote cGAS activity and cGAMP production.^[^ ^13^ ^]^ Similarly, DNA sensor IFI16 binds to viral DNA to initiate filament‐like LLPS and induces the production of antiviral cytokines.^[^ ^14^ ^]^ In contrast, overproduction of cGAMP induces the formation of the stimulator of interferon genes (STING) condensates on the endoplasmic reticulum (ER), thereby preventing overactivation of the innate immune response.^[^ ^15^ ^]^ Although the importance of phase separation during viral infection is gradually recognized, whether LLPS plays a role in HBV‐mediated innate immunity and cccDNA inhibition is still unknown.

The ATP‐binding cassette sub‐family F member 1 (ABCF1), a member of the ATP‐binding cassette (ABC) transporter family, was previously identified as a potential DNA sensor that associates with dsDNA and modulates the innate immune responses and retroviral infection.^[^ ^16^ ^]^ Unlike other ABC proteins, ABCF1 lacks a transmembrane domain and is located both in cytoplasm and nucleoplasm,^[^ ^17^ ^]^ and regulates numerous cellular processes and diseases.^[^ ^18^, ^19^, ^20^, ^21^ ^]^ ABCF1 protein contains an N‐terminal domain that interacts with eukaryotic initiation factor 2 and C terminal nucleotide‐binding domains NBD1 and NBD2 which bind and hydrolyze ATP.^[^ ^17^ ^]^ Recently, the N‐terminal domain of ABCF1 is predicted to be a highly disordered low‐complexity domain (LCD), which contributes to its interaction with SRY‐box transcription factor 2 (SOX2) and transcriptional activation of pluripotency genes in a liquid‐liquid phase separation (LLPS) manner.^[^ ^22^ ^]^ However, whether ABCF1 is involved in HBV recognition and inhibition remains elusive.

In this study, based on the whole cell‐based screening of hepatic cccDNA‐binding proteins,^[^ ^23^ ^]^ we identified ABCF1 as a novel cccDNA‐binding factor and host restriction factor for HBV replication. Mechanistically, ABCF1 senses cccDNA by KKx4 motif to form phase‐separated condensates with cccDNA via its poly‐glutamine (PolyQ) in the LCD domain, thereby activating innate immune signaling and preventing RNA polymerase II (Pol II) recruitment to cccDNA. Our findings provide a novel insight into cccDNA regulation which may contribute to the development of new strategies for HBV treatment.

## 2 Results

### 2.1 ABCF1 is Identified as a cccDNA‐Interacting Protein to Activate Innate Immune Signaling

**Figure 1:**
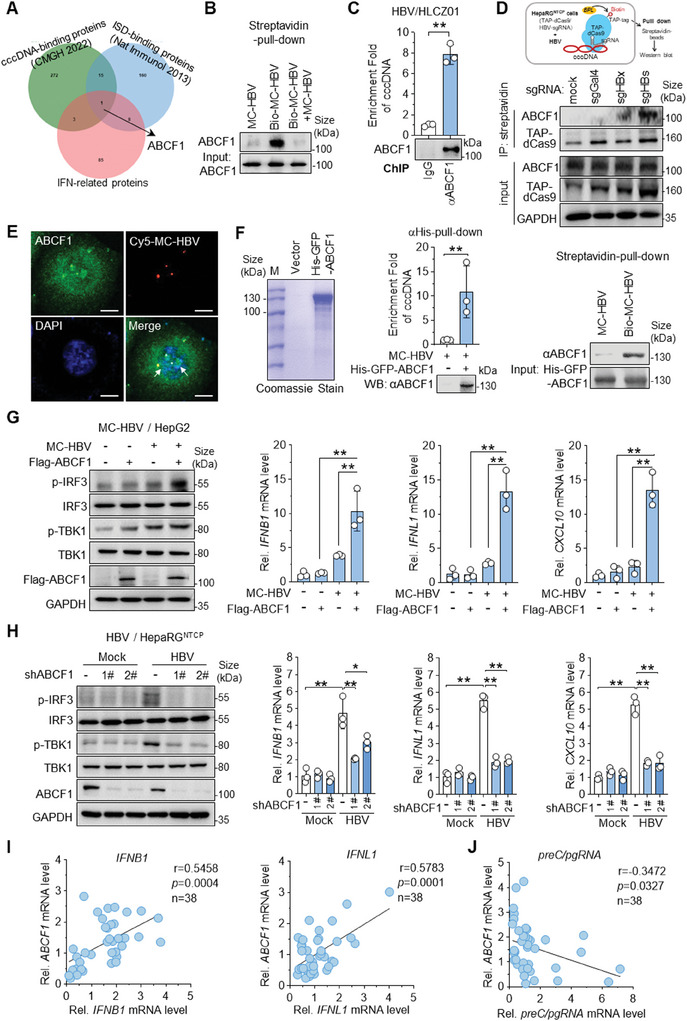
ABCF1 as a cccDNA‐binding protein to activate innate immune signaling. A) Venn analysis of cccDNA‐binding proteins, ISD‐binding proteins, and interferon‐related proteins that were adopted from previously published data.^[^ ^16^, ^23^ ^]^ ABCF1 was identified as a uniquely shared host protein among three sets. B) Pull‐down analysis of ABCF1 and cccDNA interaction. Nuclear proteins derived from HepG2 cells were incubated with biotinylated‐MC‐HBV (Bio‐MC‐HBV), unlabeled MC‐HBV used as a competitor. After streptavidin‐pull‐down, the binding of ABCF1 with MC‐HBV was evaluated by Western blot. C) ChIP analysis of ABCF1‐cccDNA interaction in HBV‐infected HLCZ01 cells. HBV infected HLCZ01 cells for 5 days, and the enrichment of ABCF1 on cccDNA was measured by ChIP‐qPCR with cccDNA‐specific primers. The efficiency of ChIP immunoprecipitation was evaluated by Western blot using anti‐ABCF1. *n* = 3 biologically independent samples. D) In vivo detection of ABCF1‐cccDNA interaction by CAPTURE (CRISPR affinity purification in situ of regulatory elements).^[^ ^26^ ^]^ HepaRG^NTCP^ cells with stable expression of biotin acceptor TAP‐tagged dCas9 and HBV‐targeted sgRNAs were infected with HBV for 7 days, and dCas9‐mediated ChIP was performed with streptavidin to identify the binding of ABCF1 with cccDNA. The dCas9 alone (mock) and HBV non‐targeting sgGal4 were taken as control. E) Co‐localization of ABCF1 and cccDNA detected by confocal microscopy. Cy5‐MC‐HBV was transfected into HepG2 cells for 72 h, ABCF1 was immunostained and the nucleus was stained with 4,6‐diamidino‐2‐phenylindole (DAPI). The representative image was shown from three independent experiments. Scale bars, 10 µm. F) In vitro binding assay with ABCF1 and cccDNA. Recombinant His‐ABCF1 protein was purified (left panel) and its binding with MC‐HBV was analyzed by αHis‐pull‐down and streptavidin‐pull‐down (right panel). *n* = 3 biologically independent samples. G,H) ABCF1 overexpression enhances (G) while ABCF1 knockdown attenuates (H) HBV‐induced IFN responses. ABCF1 was transfected with or without MC‐HBV into HepG2 cells for 48 h (G) or ABCF1‐siRNAs were transfected into HBV‐infected HepaRG^NTCP^ cells for 72 h (H). Phosphorylation of TBK1 and IRF3 was determined by Western blot, while *IFNB1*, *IFNL1*, and *CXCL10* expression were measured by RT‐qPCR. The control group “‐” was added with vector plasmid. *n* = 3 biologically independent samples. I,J) Correlation analysis of *ABCF1* expression with levels of *IFNB1*, *IFNL1* (I), and *preC/pgRNA* (J) in para‐tumor tissues from HCC patients by RT‐qPCR. *n* = 38 HCC patients. Results are representative of two (B–H) independent experiments. *p*‐Values were determined by unpaired two‐tailed t‐tests (C, F) or 1‐way analysis of variance (G, H); ^*^ *p* < 0.05, ^**^ *p* < 0.01.

To identify the candidate cccDNA sensor that is responsible for HBV inhibition, by Venn analysis, we screened the common proteins from 3 published data, including whole cell‐based cccDNA‐interaction protein profile,^[^ ^23^ ^]^ interferon‐stimulatory DNA (ISD)‐interacting proteins^[^ ^16^ ^]^ and IFN‐related signaling proteins. Intriguingly, ABCF1 was identified as a unique shared protein among three sets (**Figure** **1**A). The interaction of ABCF1 with cccDNA was verified by a pull‐down assay using HepG2 cell nuclear proteins and a biotin‐labeled cccDNA (genotype D) surrogate named MC‐HBV (Bio‐MC‐HBV).^[^ ^23^ ^]^ As shown in Figure 1B, ABCF1 was successfully pulled down by Bio‐MC‐HBV but not unlabeled free MC‐HBV, and this interaction was competed efficiently by unlabeled MC‐HBV. Consistently, cccDNA‐specific chromatin immunoprecipitation (ChIP) assay in HBV‐infected HLCZ01 cells,^[^ ^24^ ^]^ a human hepatoma cell line that supports the entire lifecycle of HBV, showed that ABCF1 significantly accumulated with cccDNA (Figure 1C), but failed to bind host genomic DNA, including *GAPDH*, *RPL30*, and *MYOD1* (Figure S1A, Supporting Information). Importantly, in HBV‐infected HepaRG^NTCP^ cells, the best surrogate model to primary human hepatocytes,^[^ ^25^ ^]^ the interaction of endogenous ABCF1 with native HBV‐derived cccDNA was also confirmed through a recently developed catalytically inactivated Cas9 (dCas9)‐based CAPTURE (CRISPR Affinity Purification in situ of Regulatory Elements) system.^[^ ^26^ ^]^ As shown in Figure 1D, HepaRG^NTCP^ cells stably expressing single guide RNA (sgRNA) targeting HBx/HBs and TAP tagged‐dCas9 were infected with HBV. The sgRNAs enable dCas9 to specifically bind to HBV DNA without affecting HBV replication (Figure S1B, Supporting Information), and TAP‐tag can be biotinylated by endogenous biotin protein ligase (BPL).^[^ ^26^ ^]^ Immunobloting assay following after ChIP with streptavidin beads showed that ABCF1 was enriched in HepaRG^NTCP^ cells expressing sgRNA targeting HBx and HBs, but not in cells expressing dCas9 alone (mock) or HBV non‐targeting sgGal4 (Figure 1D).

To further verify the ABCF1‐cccDNA interaction, we analyzed the co‐localization of ABCF1 with cccDNA in HepG2 cells. Confocal microscopy showed that endogenous ABCF1 co‐localized with Cy5‐labeled MC‐HBV (Cy5‐MC‐HBV) and formed foci‐like structures (Figure 1E). Furthermore, in vitro pull‐down assay confirmed the direct interaction of ABCF1 with cccDNA. As shown in Figure 1F, MC‐HBV was significantly enriched by purified recombinant His‐tagged ABCF1 protein, while His‐ABCF1 was pulled down by streptavidin beads after incubation with Bio‐MC‐HBV but not free MC‐HBV. The interaction of ABCF1 with cccDNA was further validated in MC‐HBV from different genotypes. As shown in Figure S1C, Supporting Information, ChIP‐qPCR demonstrated a significant accumulation of ABCF1 on cccDNA of all detected genotypes, including genotypes B, C, and D (MC‐HBV‐B, MC‐HBV‐C, and MC‐HBV‐D). All these data support that ABCF1 interacts with cccDNA either in vitro or in vivo.

Given the role of ABCF1 as a regulator of dsDNA‐mediated innate immune response,^[^ ^16^ ^]^ we thus hypothesized that ABCF1 might sense cccDNA to induce IFN response. To address this issue, ABCF1 was overexpressed in HepG2 cells with and without MC‐HBV, and the activation of IFN pathway and downstream ISG (CXCL10) expression were detected. As expected, either ABCF1 or MC‐HBV only raised IFN activation at low‐level, while ectopic ABCF1 enhanced MC‐HBV‐induced activation of types I and III IFN responses, displayed as higher phosphorylation levels of TANK binding kinase 1 (TBK1) and interferon regulatory factor 3 (IRF3), and augmented production of IFN‐β, IFN‐λ, and CXCL10 (Figure 1G). Consistently, HBV infection activated IFN pathway, while knockdown of ABCF1 significantly inhibited TBK1 and IRF3 phosphorylation, and suppressed the production of IFN‐β, IFN‐λ, and CXCL10 in HBV‐infected HepaRG^NTCP^ cells (Figure 1H). Moreover, RT‐qPCR analysis demonstrated the positive correlation between the mRNA levels of *ABCF1* and *IFNB1*, *IFNL1* in nontumor liver tissues from 38 patients with HBV‐positive HCC (Figure 1I). Collectively, these data suggest that ABCF1 interacts with cccDNA to activate IFN‐I/III response in HBV‐infected hepatocytes.

### 2.2 ABCF1 Inhibits cccDNA Transcription and HBV Replication

**Figure 2:**
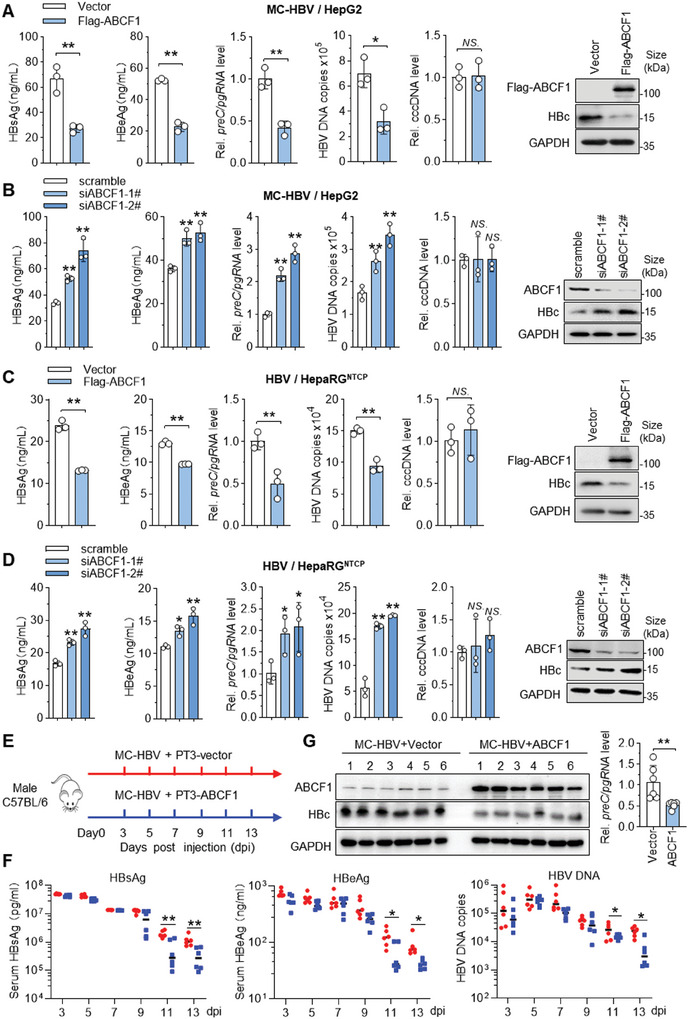
ABCF1 inhibits cccDNA transcription and HBV replication. A–D) HepG2 cells were cotransfected with MC‐HBV and Flag‐ABCF1 constructs (A) or ABCF1‐siRNAs (B) for 3 days; HepaRG^NTCP^ cells were infected with HBV at 400 Geq for 24 h and then transfected with Flag‐ABCF1 plasmids (C) or ABCF1‐siRNAs (D) for 3 days. HBsAg and HBeAg in cell culture supernatant were detected by ELISA; intracellular HBc protein levels were measured by Western blot; supernatant HBV DNA and intracellular *preC/pgRNA* were analyzed by qPCR and RT‐qPCR respectively; the extracted genomic DNA was digested with T5 exonuclease and cccDNA levels were detected by qPCR with cccDNA‐specific primers. *n* = 3 biologically independent samples. E–G) HBV carrier mice were prepared by hydrodynamic injection (HDI) of MC‐HBV with PT3‐vector or PT3‐ABCF1 plasmids (E). Blood was collected every 2 days, and mice were sacrificed at 13 days post HDI. Serum levels of HBsAg, HBeAg (ELISA), and HBV‐DNA (qPCR) were detected (F). Hepatic HBc and *preC/pgRNA* expression were measured by Western blot and RT‐qPCR, respectively (G) (*n* = 6). Results are representative of three (A–D) or two (E–G) independent experiments. *p*‐Values were determined by unpaired two‐tailed *t*‐tests; ^*^ *p *< 0.05, ^**^ *p* < 0.01, *NS*.: non‐significant.

As the aforementioned data showed that ABCF1 interacts with cccDNA to induce IFN production, and a negative correlation between *ABCF1* mRNA and precore/pregenomic RNA (*preC/pgRNA*) levels in HBV‐related human paratumor liver tissues was detected by RT‐qPCR (Figure 1J), we next investigated the effect of ABCF1 on HBV replication. As shown in **Figure** **2**A,C, ABCF1 overexpression significantly inhibited HBV replication in both MC‐HBV‐transfected HepG2 cells and HBV‐infected HepaRG^NTCP^ cells, as demonstrated by decreased levels of hepatitis B surface antigen (HBsAg), e antigen (HBeAg), core protein (HBc), *preC/pgRNA*, and HBV DNA. Consistently, ABCF1 knockdown significantly increased the levels of viral antigens (HBsAg, HBeAg, and HBc), *preC/pgRNA*, and HBV DNA (Figure 2B,D). However, the cccDNA levels were not significantly altered by either ABCF1 overexpression or ABCF1 knockdown (Figure 2A–D), suggesting that ABCF1 inhibits HBV replication at the transcriptional level.

To further confirm the role of ABCF1 against HBV in vivo, ABCF1 overexpression was introduced into HBV carrier mice by the sleeping beauty transposon system.^[^ ^27^ ^]^ Briefly, ABCF1‐PT3EF1α and MC‐HBV plasmids were hydrodynamically injected (HDI) into C57BL/6 mice (Figure 2E). As expected, ABCF1 overexpression significantly reduced the serum levels of HBsAg, HBeAg, and HBV DNA (Figure 2F), as well as hepatic *preC/pgRNA* levels and HBc expression (Figure 2G). Together, above findings demonstrate ABCF1 as a host restriction factor for cccDNA transcription and HBV replication.

### 2.3 LCD Domain Is Required for ABCF1 to Bind cccDNA and Suppress HBV Replication

**Figure 3:**
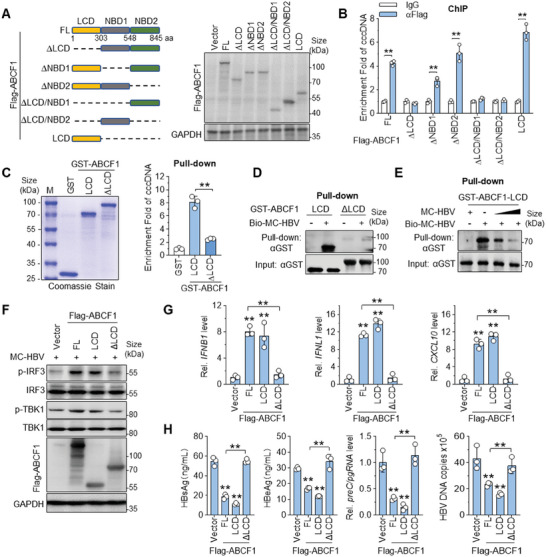
LCD domain is required for ABCF1 to bind cccDNA and suppress HBV replication. A) Schematic diagram of ABCF1 truncates (left panel) and the expression of ABCF1 and its truncates detected by Western blot (right panel). B) ChIP analysis of the interaction between ABCF1 or ABCF1 truncates and cccDNA. A series of ABCF1 truncates were cotransfected with MC‐HBV into HepG2 cells for 72 h, and the enrichment of ABCF1 truncates on cccDNA was measured by ChIP assay. *n* = 3 biologically independent samples. C,D) In vitro pull‐down assay for the interaction of ABCF1‐LCD and HBV cccDNA. Recombinant GST‐LCD and GST‐ΔLCD proteins were incubated with MC‐HBV or Bio‐MC‐HBV, and their binding was detected by GST pull‐down assay (*n* = 3 biologically independent samples) (C) and streptavidin bead pull‐down assay (D), respectively. The recombinant GST‐LCD and GST‐ΔLCD proteins were purified and detected by Coomassie stain (C left panel). E) Competitive pull‐down assay. Recombinant GST‐LCD protein was incubated with Biotin‐MC‐HBV with or without the presence of unlabeled MC‐HBV respectively, and then pull‐down assay was performed with streptavidin beads. F,G) LCD domain is required for ABCF1 to promote IFN activation upon HBV infection. ABCF1, ABCF1‐LCD, or ABCF1ΔLCD was overexpressed in MC‐HBV‐transfected HepG2 cells for 48 h. Phosphorylation of TBK1 and IRF3 was determined by Western blot (F), while *IFNB1*, *IFNL1*, and *CXCL10* expression were measured by RT‐qPCR (G). *n* = 3 biologically independent samples. H) ABCF1‐mediated HBV inhibition depends on LCD domain. ABCF1 constructs were cotransfected with MC‐HBV into HepG2 cells for 3 days. Levels of HBsAg, HBeAg (ELISA), *preC/pgRNA* (RT‐qPCR), and HBV DNA (qPCR) were measured. *n* = 3 biologically independent samples. Results are representative of two (A–E) or three (F–H) independent experiments. *p*‐Values were determined by unpaired two‐tailed *t*‐tests (B,C) or one‐way analysis of variance (G,H); *p < 0.05, **p < 0.01.

To define the mechanistic basis underlying ABCF1‐cccDNA interaction, we next mapped the vital domain of ABCF1 binding to cccDNA. ABCF1 contains an N‐terminal intrinsically disordered low‐complexity domain (LCD) and two nucleotide‐binding domains (NBD1 and NBD2).^[^ ^17^ ^]^ To identify the key domain responsible for ABCF1‐cccDNA interaction, we constructed a series of ABCF1 truncated mutants. Western blot confirmed the expression of recombinant proteins (**Figure** **3**A). Immunofluorescence staining showed similar subcellular localization of ABCF1 mutants as ABCF1‐FL (Figure S2A, Supporting Information). To compare the cccDNA binding activity, MC‐HBV were cotransfected with Flag‐ABCF1 or its different truncates into HepG2 cells for ChIP assay. As shown in Figure 3B and Figure S2B, Supporting Information, compared with full‐length ABCF1 (ABCF1‐FL), the deletion of either LCD alone (ΔLCD), or together with NBD1 (ΔLCD/NBD1) or NBD2 (ΔLCD/NBD2) largely abolished the enrichment of ABCF1 on cccDNA, while ΔNBD1 or ΔNBD2 truncation only slightly affected the enrichment, indicating that LCD is required for ABCF1 to occupy cccDNA. In accordance, LCD itself maintained a strong cccDNA‐binding ability (Figure 3B). These results illustrate that LCD is the pivotal domain determining ABCF1‐cccDNA interaction.

To further confirm this interaction, we purified recombinant GST‐ABCF1‐LCD and GST‐ABCF1‐NBD1/2 (GST‐ABCF1ΔLCD) proteins and adopted GST pull‐down assay with Bio‐MC‐HBV in vitro. As shown in Figure 3C, cccDNA surrogate MC‐HBV was strongly enriched by GST‐ABCF1‐LCD protein but not by GST‐ABCF1ΔLCD. Furthermore, streptavidin beads coupled with Bio‐MC‐HBV specifically pulled down GST‐ABCF1‐LCD protein but not GST‐ABCF1ΔLCD protein (Figure 3D), and this interaction of GST‐ABCF1‐LCD and Bio‐MC‐HBV was efficiently competed by unlabeled MC‐HBV in a dose‐dependent manner (Figure 3E). All these data suggest that ABCF1 directly binds to HBV cccDNA in an LCD‐dependent manner.

Next, we assayed whether LCD domain is also responsible for ABCF1‐mediated IFN activation during HBV infection. To address this, different ABCF1 mutants were overexpressed in MC‐HBV‐transfected HepG2 cells. As shown in Figure 3F,G, increased p‐TBK1 and p‐IRF3 levels and augmented production of IFN‐β, IFN‐λ, and CXCL10 were detected in cells with ectopic expression of ABCF1‐FL and ABCF1‐LCD, but not in cells with overexpression of ABCF1ΔLCD. Consistently, compared with ABCF1‐FL, ABCF1‐LCD but not ABCF1ΔLCD retained the ability to repress HBsAg, HBeAg, *preC/pgRNA*, and HBV DNA levels in MC‐HBV‐transfected HepG2 cells and HBV‐infected HLCZ01 cells (Figure 3H; Figure S2C, Supporting Information). All these data demonstrate that LCD is critical for ABCF1‐cccDNA binding and the consequent IFN induction and HBV inhibition.

### 2.4 ABCF1 Interacts with cccDNA to form Phase‐Separated Condensates via LCD Domain

**Figure 4:**
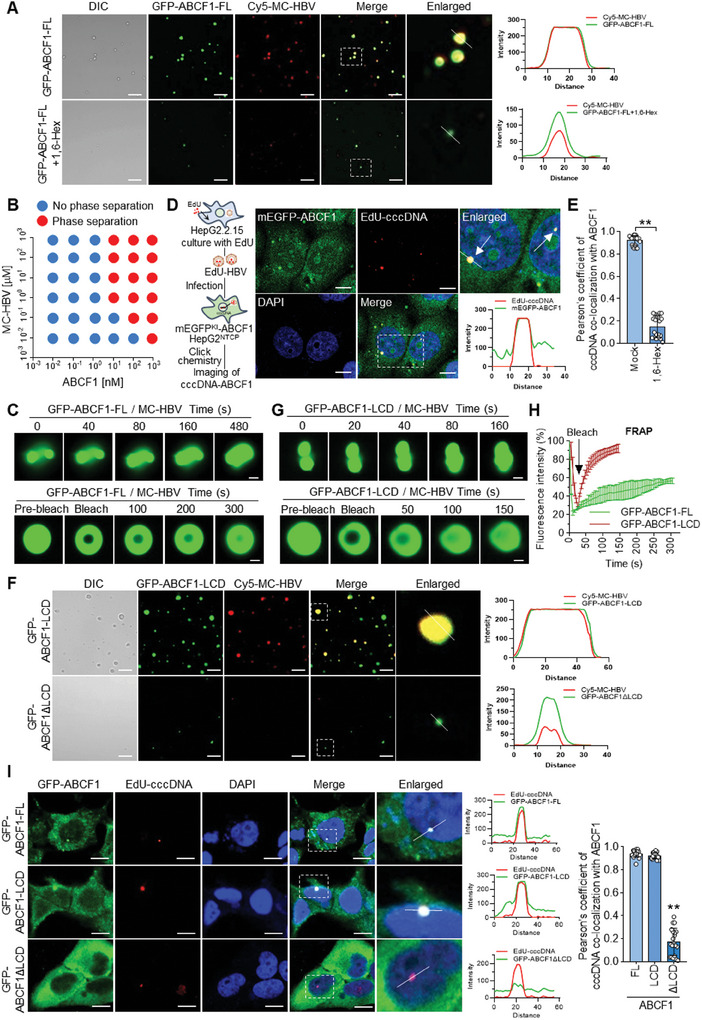
ABCF1 interacts with cccDNA to form phase‐separated condensates via LCD domain. A) Differential interference contrast (DIC) and concurrent fluorescence imaging of ABCF1‐cccDNA condensates prepared in vitro. Recombinant GFP‐ABCF1 was incubated with Cy5‐MC‐HBV for 5 min at 37 °C in 10% PEG8000 buffer with/without the presence of 10% 1,6‐Hex. The formation of liquid droplets was imaged and representative images are shown. The intensity profiles of colocalization of ABCF1 with MC‐HBV correspond to the white boxes drawn in the enlarged images. Scale bar, 10 µm. B) Phase separation diagram of ABCF1‐FL and MC‐HBV at the indicated concentrations in 10% PEG8000 buffer for 5 min at 37 °C. Red dots indicate phase separation of ABCF1‐MC‐HBV, and blue dots indicate no phase separation. C,G,H) Representative time‐lapse images (upper panel) and FRAP assay (lower panel) of ABCF1‐cccDNA liquid droplets (C) or ABCF1‐LCD‐cccDNA liquid droplets (G) that formed as described in (A). Photobleaching was performed at indicated time points, and fluorescence intensity recovery was followed and imaged in time. Scale bar, 2 µm. FRAP recovery curve of ABCF1‐FL droplets and ABCF1‐LCD droplets were fitted by a single exponential decay function (H). Data are shown as the mean ± SD (*n* = 3 droplets). D) ABCF1‐cccDNA puncta formation in live HBV‐infected HepG2^NTCP^ cells. HBV virion was metabolically labelled with EdU in HepG2.2.15 cells to infect HepG2^NTCP^ cells which carry the mEGFP‐ABCF1 knockin. After 72 h, EdU‐cccDNA was stained with Alexa Fluor 647 dye by click chemistry technique, and ABCF1‐cccDNA puncta was analyzed by confocal microscopy. The left panel shows the experimental workflow and right panel shows the representative images. The white box represents the enlarged region. Scale bars, 10 µm. E) The Pearson's coefficient of cccDNA colocalization with ABCF1 in live HBV‐infected HepG2^NTCP^ cells with or without 1,6‐Hex treatment. F) DIC and concurrent fluorescence imaging of LLPS formed by Cy5‐MC‐HBV and ABCF1‐LCD (up panel) or ABCF1ΔLCD (low panel) in vitro. Recombinant truncated ABCF1 proteins (GFP‐ABCF1‐LCD and GFP‐ABCF1ΔLCD) and Cy5‐MC‐HBV were incubated in 10% PEG8000 buffer for 5 min at 37 °C. The formation of liquid droplets was imaged and representative images are shown. The intensity profiles correspond to the white lines drawn in the enlarged images. Scale bar, 10 µm. I) Immunofluorescence assay of LCD‐cccDNA puncta formation in HepG2^NTCP^ cells. HepG2^NTCP^ cells were infected with EdU‐labeled HBV at 400 Geq for 24 h and then transfected with GFP‐ABCF1, GFP‐ABCF1‐LCD, or GFP‐ABCF1ΔLCD plasmids for 2 days, and the puncta were imaged by confocal microscopy and their colocalization was analyzed by the Pearson's coefficient (right panel). Scale bars, 10 µm. Results are representative of three (A‐I) independent experiments. *p*‐Values were determined by unpaired two‐tailed t‐tests (E,I); **p < 0.01.

A recent study reported that ABCF1 contributes to phase separation which plays a crucial role in innate immune signaling.^[^ ^13^, ^15^, ^22^ ^]^ Intriguingly, we observed that ABCF1 colocalized with MC‐HBV and formed foci‐like structures (Figure 1E). Thus, we are wondering whether ABCF1 forms condensate with HBV cccDNA. To test this, we incubated monomeric enhanced green fluorescent protein (mEGFP)‐fused full‐length ABCF1 protein (GFP‐ABCF1‐FL) with Cy5‐MC‐HBV in 10% PEG8000 reaction buffer that mimics the intracellular crowding environment,^[^ ^28^, ^29^ ^]^ and analyzed the formation of liquid droplets in vitro. As shown in **Figure** **4**A, upon mixed, ABCF1‐FL and MC‐HBV formed micrometer‐sized liquid droplets within 5 min, which was substantially inhibited by 10% 1,6‐hexanediol (1,6‐Hex), a commonly used LLPS inhibitor.^[^ ^30^, ^31^ ^]^ Consistently, ABCF1 and cccDNA also formed micrometer‐sized liquid droplets in a physiological buffer,^[^ ^13^ ^]^ NaCl buffer, 2.5% PEG8000 buffer, 20% BSA buffer, and 20% Ficoll buffer (Figure S3A, Supporting Information). With the increase of MC‐HBV, ABCF1‐cccDNA liquid‐like droplets formed at lower concentrations, indicating that MC‐HBV accelerated the formation of ABCF1‐cccDNA LLPS (Figure 4B). Notably, time‐lapse microscopy observed fusion events between ABCF1 liquid droplets (Figure 4C, upper panel). Fluorescence recovery after photobleaching (FRAP) experiments showed that after bleaching, about 50% of ABCF1‐FL fluorescence signals were efficiently recovered 200 s later (Figure 4C (lower panel),H).

To verify the ABCF1‐cccDNA phase separation within HBV‐infected cells, mEGFP‐ABCF1 knockin (mEGFP^KI^‐ABCF1) HepG2^NTCP^ cells were prepared (Figure S3B, Supporting Information), thus endogenous ABCF1 can be traced by monomeric GFP in vivo. To trace cccDNA in cells, 5′‐ethynyl‐2′‐deoxyuridine (EdU) was added into HepG2.2.15 cell culturing, allowing the incorporation of EdU into relaxed circular DNA (RC‐DNA) of nascent HBV virion. The EdU‐HBV virions were then used to infect NTCP‐expressed Huh7 (Huh7^NTCP^) cells, in which RC‐DNA was repaired into cccDNA in the nucleus and can be labeled by copper‐catalyzed azide‐alkyne cycloaddition. As shown in Figure S3C,D, Supporting Information, EdU‐cccDNA was only detected in EdU‐HBV‐infected Huh7^NTCP^ cells, but not in HBV‐infected Huh7^NTCP^ cells, and EdU‐HBV exhibited comparable replication levels with unlabeled HBV. Furthermore, in EdU‐HBV‐infected mEGFP^KI^‐ABCF1‐HepG2^NTCP^ cells, confocal microscopy showed that endogenous mEGFP‐ABCF1 formed a foci‐like structure with EdU‐cccDNA in the nucleus (Figure 4D). Quantification assay of ABCF1‐cccDNA colocalization showed that the majority of cccDNA overlapped with ABCF1 and this colocalization was largely eliminated upon 1,6‐Hex treatment (Figure 4E; Figure S3E, Supporting Information). These data support that HBV cccDNA forms liquid phase condensation with ABCF1.

Next, to address whether LCD is responsible for the phase separation of ABCF1 with cccDNA, we incubated Cy5‐MC‐HBV with recombinant GFP‐ABCF1‐LCD and GFP‐ABCF1ΔLCD respectively, the purity of recombinant proteins was verified by Coomassie staining (Figure S3F, Supporting Information). As expected, the liquid droplets fused with Cy5‐MC‐HBV were only formed by GFP‐ABCF1‐LCD but not by GFP‐ABCF1ΔLCD protein (Figure 4F). Additionally, time‐lapse microscopy observed the fusion and faster fluorescence recovery after FRAP of GFP‐ABCF1‐LCD droplets than that of ABCF1‐FL droplets after mixing with MC‐HBV, about 80% of ABCF1‐LCD fluorescence signals were recovered at 50 s after photobleaching (Figure 4G,H). To verify the LCD‐dependent ABCF1‐cccDNA LLPS in cells, HepG2^NTCP^ cells were infected with EdU‐labeled HBV, and then transfected with GFP‐ABCF1‐FL, LCD, or ΔLCD respectively. As shown in Figure 4I, EdU‐cccDNA formed condensates with ABCF1‐FL or ABCF1‐LCD but not ABCF1ΔLCD in the nucleus. Together, these data suggest that ABCF1 interacts with cccDNA to drive liquid‐like behavior in an LCD‐dependent fashion.

### 2.5 ABCF1‐LCD Senses cccDNA and Inhibits HBV Replication Through Phase Separation

**Figure 5:**
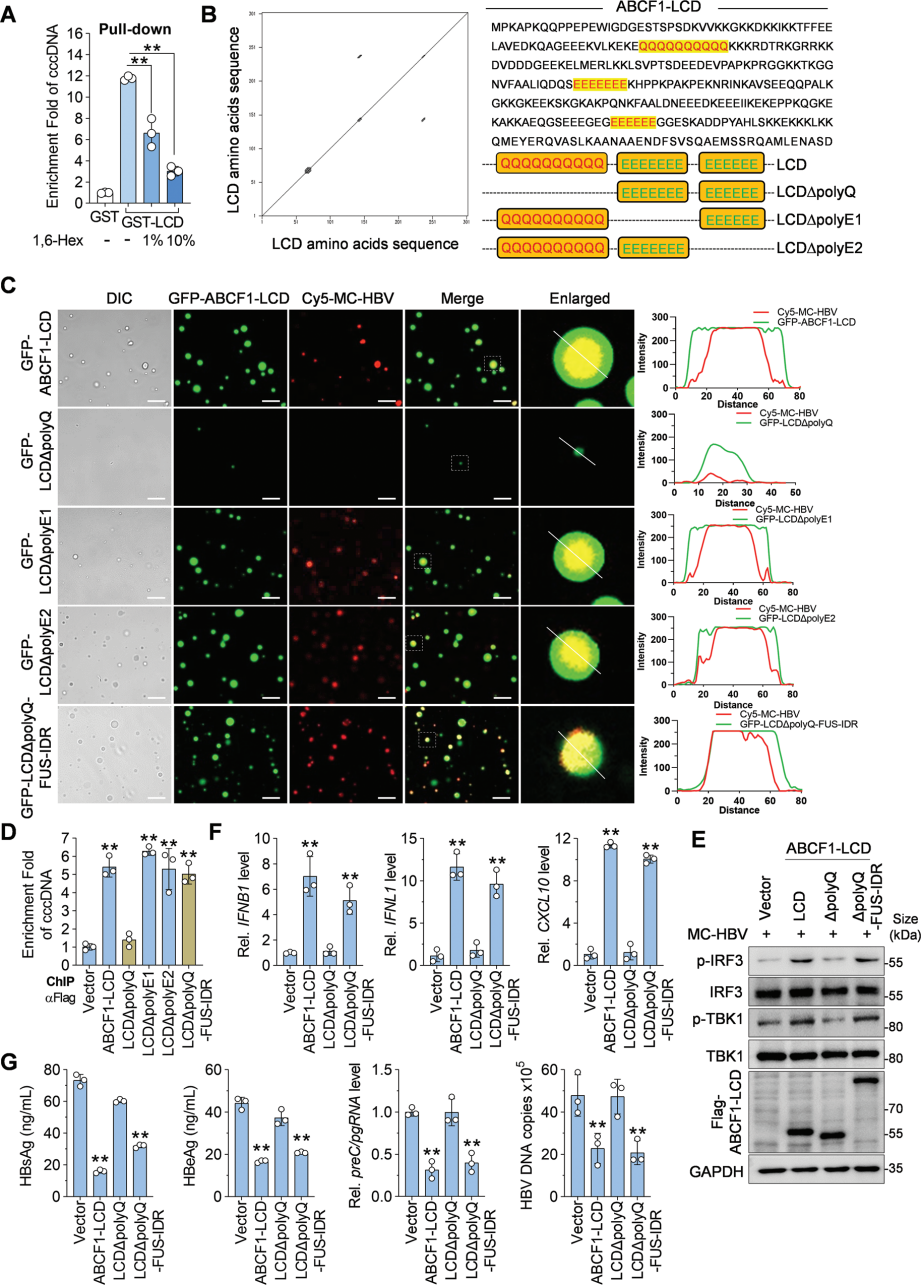
LCD‐polyQ mediated LLPS is required for ABCF1‐LCD‐promoted IFN response and HBV inhibition. A) GST pull‐down assay showing the interaction of LCD and cccDNA. GST‐ABCF1‐LCD protein and MC‐HBV were coincubated for 8 h in 10% PEG8000 buffer with or without 1,6‐Hex, and their binding was analyzed by GST pull‐down assay. *n* = 3 biologically independent samples. B) Repeat amino acid sequence analysis of ABCF1‐LCD by Dottup tool and the schematic of ABCF1‐LCD mutants with repeat amino acid deletion. C) Phase separation of ABCF1‐LCD mutants with cccDNA. Recombinant GFP‐LCD, LCDΔpolyQ, LCDΔpolyE1, LCDΔpolyE2, and LCDΔpolyQ‐FUS‐IDR were respectively incubated with Cy5‐MC‐HBV for 5 min at 37 °C in 10% PEG8000 buffer and the formation of liquid droplets was imaged. The intensity profiles correspond to the white lines drawn in the enlarged images. Scale bar, 10 µm. D–G) Phase separation is crucial for ABCF1‐cccDNA interaction, HBV‐mediated IFN activation, and ABCF1‐mediated repression on HBV replication. ABCF1‐LCD or its mutants were cotransfected with MC‐HBV into HepG2 cells for 3 days, and the enrichment of ABCF1‐LCD on cccDNA was measured by ChIP‐qPCR (D); the phosphorylation levels of TBK1 and IRF3 at 48 h post‐transfection were determined by Western blot (E) and *IFNB1*, *IFNL1*, and *CXCL10* expression were measured by RT‐qPCR (F); viral HBsAg, HBeAg (ELISA), *preC/pgRNA* (RT‐qPCR), and HBV DNA levels (qPCR) were measured (G). *n* = 3 biologically independent samples. Results are representative of three (A,C–G) independent experiments. *p*‐Values were determined by one‐way analysis of variance (A,D,F,G); **p < 0.01.

Given the role of ABCF1‐LCD in determining ABCF1‐cccDNA condensates formation as well as in enhancing ABCF1‐mediated IFN induction and HBV inhibition, we next sought to address whether LCD interacts with cccDNA to regulate HBV replication by phase separation. Results of in vitro pull‐down assay showed that MC‐HBV was efficiently enriched by GST‐LCD protein, and this enrichment was significantly inhibited by 1,6‐Hex in a dose‐dependent manner (**Figure** **5**A). Intriguingly, 1,6‐Hex treatment either for 2 h or 4 h, which showed no significant cytotoxicity, damaged ABCF1‐FL and ABCF1‐LCD‐mediated inhibition on *preC/pgRNA* production in both MC‐HBV‐transfected HepG2 cells and HBV‐infected HLCZ01 cells (Figure S4A,B, Supporting Information). These data indicated that LLPS is required for ABCF1‐LCD to bind cccDNA and inhibit its transcription.

To exclude the non‐specific effects of 1,6‐Hex, we further defined the critical amino acids that determine the formation of ABCF1‐cccDNA droplets. Some special amino acid repeats, such as glutamine or alanine repeats, have been reported to contribute to condensate formation.^[^ ^32^ ^]^ Dottup analysis with EMBOSS program (https://www.ebi.ac.uk/Tools/emboss/) found three tandem amino acid repeats regions featured with poly‐glutamine (polyQ) or poly‐glutamic acid (polyE) in ABCF1‐LCD (Figure 5B). LLPS assays in vitro showed that ABCF1‐LCD‐cccDNA phase transition was largely obstructed by the deletion of polyQ domain (LCDΔpolyQ) but not by the deletion of polyE1 (LCDΔpolyE1) or polyE2 (LCDΔpolyE2) (Figure 5C; Figure S4C, Supporting Information). This was further verified in live HBV‐infected cells. As shown in Figure S4D, Supporting Information, ABCF1‐LCD formed puncta with cccDNA in EdU‐HBV‐infected HepG2^NTCP^ cells, while LCD‐cccDNA foci were not observed in LCDΔpolyQ‐expressing cells. These data suggest that ABCF1 forms LLPS with cccDNA via polyQ in the LCD domain.

Next, to determine whether ABCF1‐cccDNA phase transition is responsible for IFN activation and HBV inhibition, we introduced another LLPS‐prone intrinsically disordered regions (IDRs) of FUS protein (FUS‐IDR)^[^ ^33^ ^]^ into LCDΔpolyQ mutant (LCDΔpolyQ‐FUS‐IDR). As shown in Figure 5C, recombinant LCDΔpolyQ‐FUS‐IDR formed liquid‐like droplets with Cy5‐MC‐HBV at a comparable level as LCD, which is in line with the previous study.^[^ ^33^ ^]^ Importantly, ChIP analysis showed that the enrichment of ABCF1‐LCD on cccDNA was damaged by polyQ deletion, and rescued by FUS‐IDR addition into LCDΔpolyQ (Figure 5D; Figure S4E, Supporting Information). Notably, ABCF1‐LCD‐mediated increase of p‐TBK1 and p‐IRF3 levels and IFN‐β, IFN‐λ, and CXCL10 production were significantly weakened by LCDΔpolyQ but recovered by LCDΔpolyQ‐FUS‐IDR in MC‐HBV‐transfected HepG2 cells (Figure 5E,F). In accordance, ABCF1‐LCD and LCDΔpolyQ‐FUS‐IDR but not LCDΔpolyQ inhibited the levels of HBsAg, HBeAg, *preC/pgRNA*, and HBV DNA in MC‐HBV‐transfected HepG2 cells and HBV‐infected HLCZ01 cells (Figure 5G; Figure S4F, Supporting Information). Together, these data imply that the formation of ABCF1‐LCD‐cccDNA condensates is essential for ABCF1‐mediated IFN activation and HBV replication inhibition.

### 2.6 KKx4 Motif of ABCF1‐LCD Dominated cccDNA‐Binding Contributes to LLPS

**Figure 6:**
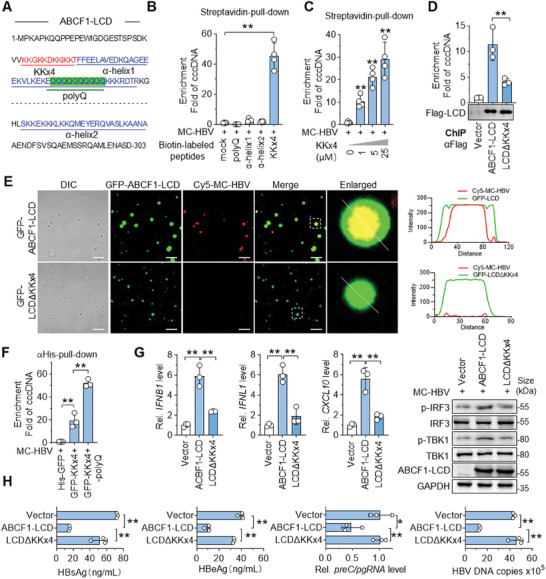
KKx4 motif of LCD is essential for ABCF1‐cccDNA interaction and its antiviral role. A) Sequences and location of KKx4, polyQ, α‐helix1, and α‐helix2 in ABCF1‐LCD. B) In vitro binding assays of different peptides with cccDNA. Biotin‐labeled peptides that contain the polyQ, α‐helix1, α‐helix2, or KKx4 were incubated with MC‐HBV respectively, and their binding with cccDNA was analyzed by streptavidin‐pull‐down. *n* = 4 biologically independent samples. C) Pull‐down assay of Biotin‐KKx4 at different doses with cccDNA was performed as Figure 6B. *n* = 4 biologically independent samples. D) ChIP analysis of the interaction of ABCF1‐LCDΔKKx4 with cccDNA as Figure 3B, the efficiency of ChIP immunoprecipitation was evaluated by Western blot using anti‐Flag. *n* = 3 biologically independent samples. E) DIC and concurrent fluorescence imaging of LLPS formed by Cy5‐MC‐HBV and recombinant GFP‐ABCF1‐LCD or GFP‐LCDΔKKx4 in vitro as Figure 4A. Scale bar, 10 µm. F) In vitro pull‐down analysis of GFP‐KKx4 and cccDNA interaction. Recombinant His‐GFP, His‐GFP‐KKx4, or His‐GFP‐KKx4‐polyQ proteins were incubated with MC‐HBV, and their interaction was analyzed by αHis‐pull‐down assay (*n* = 3 biologically independent samples). G,H) The role of KKx4 in regulating ABCF1‐mediated IFN activation and antiviral effect upon HBV infection. ABCF1‐LCD or LCDΔKKx4 were cotransfected with MC‐HBV into HepG2 cells for 2 (G) or 3 (H) days. The phosphorylation levels of TBK1 and IRF3 were detected by Western blot and the mRNA levels of *IFNB1*, *IFNL1*, and *CXCL10* were determined by RT‐qPCR, respectively (G); viral HBsAg, HBeAg, *preC/pgRNA*, and HBV DNA levels were measured by ELISA and qPCR, respectively (H). *n* = 3 biologically independent samples. Results are representative of three (B–H) independent experiments. *p*‐values were determined by unpaired two‐tailed *t*‐tests; *p < 0.05, **p < 0.01.

To sense cccDNA is a prerequisite for the formation of ABCF1‐cccDNA condensates, we next asked whether polyQ is also responsible for cccDNA recognition. However, MC‐HBV was not pulled down by biotin‐labeled polyQ peptides (Figure 6B), suggesting there are other regions that involve cccDNA‐binding. The positively charged residues (Arg and Lys)‐rich domain and the α‐helix structural elements are most commonly observed in DNA‐binding proteins.^[^ ^34^, ^35^ ^]^ Upon analysis of ABCF1 amino acid composition and protein structure based on the AlphaFold Protein Structure Database, two α‐helix elements (α‐helix1 and α‐helix2) and one Lys‐rich domain (KKGKKDKKIKKT, named KKx4) were found in ABCF1‐LCD domain (**Figure** **6**A). Further pull‐down assay verified that biotin‐labeled KKx4 peptide, but not biotin‐labeled α‐helix1 and α‐helix2 peptides, efficiently captured MC‐HBV in a dose‐dependently manner (Figure 6B,C). Consistently, KKx4 deletion in ABCF1‐LCD led to a significant decrease in the enrichment of ABCF1‐LCD on cccDNA by ChIP analysis (Figure 6D). More importantly, LLPS in vitro assays showed that KKx4 deletion (GFP‐LCDΔKKx4) obviously attenuated the phase transition of GFP‐ABCF1‐LCD with Cy5‐MC‐HBV. Specifically, fewer Cy5‐MC‐HBV were found in their droplets and the droplets were smaller (Figure 6E; Figure S5A, Supporting Information). Consistently, in EdU‐HBV‐infected HepG2^NTCP^ cells, ABCF1‐LCD‐cccDNA puncta were not observed in LCDΔKKx4‐expressing cells (Figure S5B, Supporting Information). Specifically, the pull‐down assay showed that compared with recombinant GFP‐KKx4 protein, the protein fused with polyQ and KKx4 (KKx4‐polyQ) displayed significantly higher enrichment on cccDNA (Figure 6F). LLPS assays also showed that KKx4‐polyQ peptide fused with mEGFP (GFP‐KKx4‐polyQ) was enough to form droplets with Cy5‐MC‐HBV in vitro (Figure S5C, Supporting Information). All these results suggested that the KKx4 motif is responsible for cccDNA sensing of ABCF1‐LCD and polyQ‐mediated phase separation in turn facilitates the interaction of ABCF1‐LCD with cccDNA.

As the aforementioned data clearly demonstrated that ABCF1‐cccDNA LLPS is important for IFN activation and transcription inhibition of cccDNA. This is further supported by the increased p‐TBK1 and p‐IRF3 levels and augmented production of IFN‐β, IFN‐λ, and CXCL10 in MC‐HBV‐transfected HepG2 cells with ectopic expression of ABCF1‐LCD, but not in cells with overexpression of LCDΔKKx4 (Figure 6G). Consistently, compared with ABCF1‐LCD, LCDΔKKx4 lost the ability to repress HBsAg, HBeAg, *preC/pgRNA*, and HBV DNA levels in both MC‐HBV‐transfected HepG2 cells and HBV‐infected HLCZ01 cells (Figure 6H; Figure S5D, Supporting Information). Collectively, all these data demonstrate that KKx4‐mediated cccDNA sensing is critical for the formation of ABCF1‐cccDNA condensates and the consequent IFN induction.

### 2.7 ABCF1 Phase Separation Prevents cccDNA from Transcriptional Machinery

**Figure 7:**
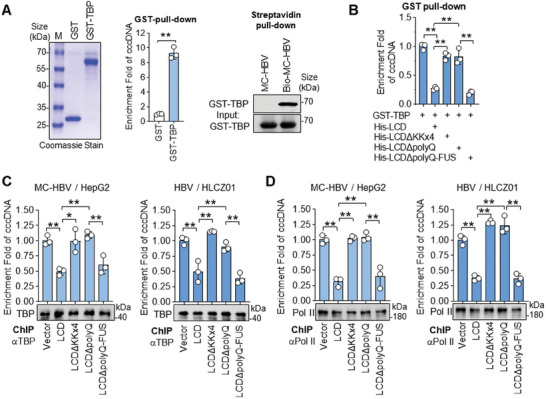
ABCF1 reduces the accumulation of Pol II and TBP on cccDNA in a phase separation‐dependent manner. A) In vitro pull‐down analysis of TBP‐cccDNA interaction. Recombinant GST‐TBP was incubated with MC‐HBV or Bio‐MC‐HBV, and their binding was detected by GST pull‐down assay (middle panel, *n* = 3 biologically independent samples) and streptavidin bead pull‐down assay (right panel), respectively. The recombinant GST‐TBP proteins were purified and detected by Coomassie stain (left panel). B) ABCF1 suppresses TBP recruitment to cccDNA in an LLPS‐dependent manner. Recombinant His‐ABCF1‐LCD, His‐LCDΔKKx4, His‐LCDΔpolyQ, or His‐LCDΔpolyQ‐FUS‐IDR proteins were incubated with MC‐HBV for 10 min, followed by adding GST‐TBP proteins for another 2 h, and the binding GST‐TBP with cccDNA was detected by GST pull‐down assay. *n* = 3 biologically independent samples. C,D) Phase separation is required for ABCF1‐mediated inhibition of TBP and Pol II occupancy on cccDNA. ABCF1‐LCD, LCDΔKKx4, LCDΔpolyQ, and LCDΔpolyQ‐FUS‐IDR were overexpressed in MC‐HBV‐transfected HepG2 or HBV‐infected HLCZ01 models for 3 days, the enrichment of TBP (C) and Pol II (D) on cccDNA was analyzed by ChIP assay, and the efficiency of ChIP immunoprecipitation was evaluated by Western blot using anti‐TBP or anti‐Pol II. *n* = 3 biologically independent samples. Results are representative of three (A–D) independent experiments. *p*‐Values were determined by unpaired two‐tailed *t*‐tests (A) or one‐way analysis of variance (B–D); *p < 0.05, **p < 0.01.

LLPS has been reported to play critical roles in inhibiting activities by molecule sequestration.^[^ ^12^ ^]^ Considering that ABCF1 suppresses cccDNA transcription (Figure 2) and it is well known that enrichment of Pol II is essential for eukaryotic transcription,^[^ ^36^ ^]^ we thus hypothesized that ABCF1‐LCD prevents Pol II from loading onto cccDNA in an LLPS‐dependent manner. TATA box‐binding protein (TBP) is a general transcription factor responsible for DNA binding, Pol II recruitment, and formation of transcription initiation complexes.^[^ ^37^ ^]^ We therefore evaluated the role of ABCF1‐LCD and its LLPS‐forming ability in TBP‐cccDNA interaction. To address this, in vitro binding assays were performed with purified GST‐TBP protein and free MC‐HBV or Bio‐MC‐HBV. As shown in **Figure** **7**A, both GST pull‐down and streptavidin‐pull‐down detected the binding of TBP with cccDNA. Furthermore, competitive pull‐down assay found that the addition of His‐ABCF1‐LCD protein but not LCDΔKKx4 or LCDΔpolyQ protein significantly reduced the enrichment of TBP on cccDNA (Figure 7B). Intriguingly, His‐LCDΔpolyQ‐FUS‐IDR protein, which lacks polyQ domain but contains the ability to form LLPS, inhibited TBP accumulation on cccDNA at a comparable level as ABCF1‐LCD (Figure 7B), supporting our hypothesis that ABCF1‐cccDNA phase separation prevents TBP loading on cccDNA.

To verify this hypothesis in live cells, we performed ChIP assays in MC‐HBV‐transfected HepG2 and HBV‐infected HLCZ01 cells. As shown in Figure 7C and Figure S6A (Supporting Information), knockdown of ABCF1 promoted while overexpression of ABCF1‐LCD significantly inhibited the occupancy of TBP on cccDNA. The inhibition of TBP‐cccDNA interaction was maintained in cells with LCDΔpolyQ‐FUS‐IDR overexpression but not in cells with ectopic expression of LCD mutants that lost KKx4 (LCDΔKKx4) or polyQ (LCDΔpolyQ). In accordance, enforced expression of ABCF1‐LCD but not LCDΔKKx4 or LCDΔpolyQ significantly reduced the enrichment of Pol II on cccDNA in both MC‐HBV‐transfected HepG2 and HBV‐infected HLCZ01 cells, while LCDΔpolyQ‐FUS‐IDR restored the deficiency of LCDΔpolyQ in inhibiting Pol II occupancy on cccDNA (Figure 7D). Further coimmunoprecipitation (Co‐IP) assay did not detect the interaction between ABCF1 and TBP or Pol II (Figure S6B,C, Supporting Information), ruling out the possibility that ABCF1 inhibits TBP/Pol II loading on cccDNA through competitive binding.

RNA polymerase II works alongside general transcription factors (TFs) to assemble transcriptional machinery at the transcription start site, open the promoter DNA, and initiate gene transcription.^[^ ^38^ ^]^ Consisting with the notion that TFs, such as CCAAT enhancer‐binding protein alpha (C/EBPα) and hepatocyte nuclear factor 1 homeobox alpha (HNF1α), are important for the loading of Pol II and the transcriptional activity of cccDNA.^[^ ^39^ ^]^ TBP and Pol II highly accumulated on HBV promoters, including CP, SP1, SP2, and XP, but not on the HBsAg‐coding gene. Ectopic ABCF1 expression led to a more significant reduction of Pol II and TBP enrichment on CP, SP1, SP2, and XP (Figure S6D, Supporting Information). ChIP assay also showed that ectopic expression of ABCF1‐LCD and LCDΔpolyQ‐FUS‐IDR but not LCDΔpolyQ significantly reduced the occupancy of C/EBPα and HNF1α on cccDNA (Figure S6E, Supporting Information). All these data reveal that ABCF1 suppresses the loading of Pol II transcriptional machinery on cccDNA by KKx4 and polyQ‐mediated phase separation, or in another word, KKx4 and polyQ of ABCF1 formed condensates with cccDNA to prevent the accumulation of Pol II machinery on cccDNA.

### 2.8 HBx Transcriptionally Inhibits ABCF1 Expression

**Figure 8:**
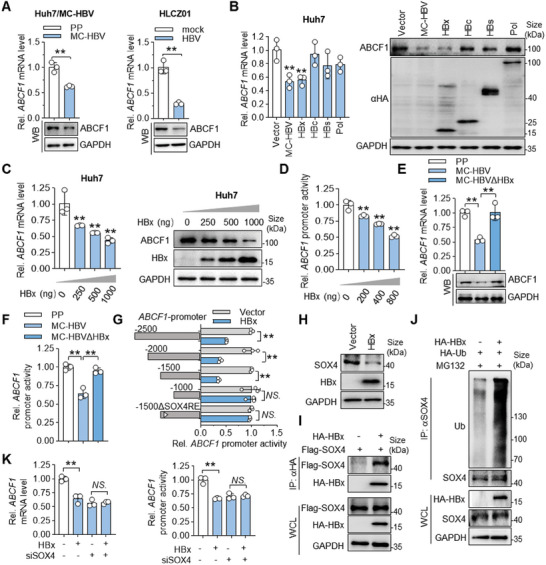
HBx transcriptionally represses ABCF1 expression. A) Detection of ABCF1 expression in different HBV replication models. Huh7 cells were transfected with MC‐HBV for 3 days (pmini‐HBV parent plasmid (PP) as control), HLCZ01 cells were infected with HBV for 5 days, and the mRNA and protein levels of ABCF1 were detected by RT‐qPCR and Western blot, respectively. *n* = 3 biologically independent samples. B) Analysis of ABCF1 expression upon ectopic expression of HBV proteins. MC‐HBV and different viral protein constructs were transfected into Huh7 cells for 3 days, and the mRNA levels of ABCF1 were measured by RT‐qPCR. Protein levels of ABCF1 and HBV viral proteins were detected by Western blots (Right panel). *n* = 3 biologically independent samples. C) ABCF1 mRNA level and protein expression were analyzed upon HBx overexpression at different doses. HA‐HBx plasmid was transfected at the indicated dose into Huh7 cells for 48 h, ABCF1 expression was analyzed as (A). *n* = 3 biologically independent samples. D) Measure of ABCF1 promoter activity upon HBx overexpression. Huh7 cells were cotransfected with ABCF1 promoter‐reporter plasmid with HA‐HBx plasmid at indicated dose. Forty‐eight hours later, the dual‐luciferase assay was performed to detect ABCF1 promoter activity. *n* = 3 biologically independent samples. E,F) Effects of HBx deletion in MC‐HBV on ABCF1 transcription. MC‐HBV and the HBx deletion (MC‐HBVΔHBx) plasmids were transfected with (F) or without ABCF1‐promoter reporter plasmid (E) into Huh7 cells for 72 hours, and ABCF1 expression (E) and promoter activity (F) were detected as (A,D), respectively. *n* = 3 biologically independent samples. (G) Identification of the core region of ABCF1 promoter regulated by HBx. HBx and a series of truncated ABCF1 promoter reporter plasmids were cotransfected into Huh7 cells for 48 h, the dual‐luciferase assay was performed to detect truncated ABCF1 promoter activity, the promoter activity of empty vector‐transfected group (vector) as 1 (right side). The left side (dark gray bars) showed the luciferase activity of different ABCF1 promoters, including the wild type ‐2000 promoter, ‐1500 promoter, and the ‐1500ΔSOX4RE promoter. *n* = 3 biologically independent samples. H) HBx reduces SOX4 protein expression. HBx plasmid was transfected into Huh7 cells for 48 hours, the protein levels of SOX4 were detected by Western blot. I) The interaction between HBx and SOX4. HA‐HBx and Flag‐SOX4 plasmids were co‐transfected into HEK293T cells for 48 h and Co‐IP assay was performed to analyze the interaction of HBx with SOX4. J) HBx promotes the ubiquitination of SOX4. Huh7 cells were cotransfected with HBx and HA‐Ub plasmids for 48 h, and then treated with MG132 for 6 h. Endogenous SOX4 was immunoprecipitated and immunoblotted with anti‐Ub antibody to measure its ubiquitylation. K) HBx inhibits ABCF1 transcription through the transcription factor SOX4. Huh7 cells were transfected with SOX4‐siRNA for 24 h and then cotransfected with ABCF1 promoter reporter and HA‐HBx plasmids for 48 h. The ABCF1 mRNA levels were measured by RT‐qPCR and ABCF1 promoter activity was detected by dual‐luciferase assay. *n* = 3 biologically independent samples. Results are representative of three (A–K) independent experiments. *p*‐Values were determined by unpaired two‐tailed *t*‐tests; *p < 0.05, **p < 0.01, *NS*.: non‐significant.

It is well known that HBV has evolved multiple mechanisms against host restriction.^[^ ^5^ ^]^ Since a negative correlation between *ABCF1* and *preC/pgRNA* levels was found in HBV‐related human paratumor liver tissues (Figure 1J), we then further investigated whether HBV regulates ABCF1 expression. As shown in **Figure** **8**A, the mRNA and protein levels of ABCF1 were downregulated in both MC‐HBV‐transfected Huh7 cells and HBV‐infected HLCZ01 cells, suggesting that HBV transcriptionally represses ABCF1 expression. To figure out how HBV regulates ABCF1 transcription, HBV proteins (HBx, HBc, L‐HBsAg, and Pol) were overexpressed in Huh7 cells. Among them, HBx rather than other viral proteins significantly repressed ABCF1 transcription and expression (Figure 8B), and HBx inhibits ABCF1 expression in a dose‐dependent manner (Figure 8C). Consistently, the luciferase reporter assay found that HBx dose‐dependently suppressed ABCF1 promoter activity (Figure 8D). Furthermore, mutation of A at 1376 (ATG→TTG) causing HBx deletion in MC‐HBV (MC‐HBV∆HBx) damaged the HBV‐mediated suppression of ABCF1 expression and its promoter activity (Figure 8E,F). Together, these results confirm the critical role of HBx in inhibiting ABCF1 transcription and expression.

To further clarify the regulatory mechanism of HBx‐mediated inhibition of ABCF1 transcription, ABCF1 promoter was sequentially truncated. Results of luciferase reporter assay showed that the deletion of the −1500 to −1000 bp region eliminated the HBx‐induced inhibition of ABCF1 promoter activity (Figure 8G). We next searched the TF‐binding motifs in ABCF1 promoter by JASPAR database (https://jaspar.elixir.no/), and a binding motif of transcription factor SOX4 (AAACAAAGGA, named SOX4RE) specifically existed in −1500 to −1000 bp region of ABCF1 promoter. Results of luciferase reporter assay and western blot showed that SOX4 transcriptionally activated ABCF1 expression in a SOX4RE‐dependent manner (Figure S7A,B, Supporting Information). Further deletion of SOX4‐responsive element in this ABCF1 promoter (‐1500ΔSOX4RE) disrupted the HBx‐mediated inhibition on ABCF1 promoter (Figure 8G). Interestingly, HBx inhibited SOX4 protein expression without affecting its transcription levels (Figure 8H; Figure S7C, Supporting Information). Co‐IP assay found that HBx interacted with SOX4, thereby inducing poly‐ubiquitination and degradation of SOX4 (Figure 8I,J; Figure S7D–F, Supporting Information). In accordance, knockdown of SOX4 abrogated HBx‐mediated suppression of ABCF1 transcription and promoter activity (Figure 8K; Figure S7G, Supporting Information). Collectively, these data suggest that HBx transcriptionally inhibits ABCF1 expression in a SOX4‐dependent manner.

## 3 Discussion

HBV cccDNA is the key barrier to HBV cure.^[^ ^2^ ^]^ Identification of host restriction factors targeting cccDNA is of great importance for the development of novel therapeutic approaches against HBV. Here, by our previously established cccDNA‐hepatocytes interactome profile,^[^ ^23^ ^]^ we identified ABCF1 as a novel cccDNA‐binding protein that inhibits HBV replication. Notably, ABCF1 interacts with cccDNA by KKx4 motif to trigger phase separation via the PolyQ of its LCD domain, thereby promoting the HBV‐mediated activation of type I/III IFN pathway and preventing cccDNA from Pol II transcriptional machinery to silence cccDNA transcription. Conversely, HBV antagonizes its antiviral effect through HBx‐mediated ABCF1 transcriptional repression, thereby escaping the inhibition of ABCF1. Thus, our data for the first time identify ABCF1 as an HBV restriction factor and provide evidence showing the involvement of LLPS in cccDNA inhibition.

Sensing HBV components by host PRRs is an essential event for the activation of innate immune responses. It has been reported that HBV RNAs can be sensed by TLR3, RIG‐I, and MDA5^[^ ^6^, ^7^, ^40^ ^]^ and HBV DNA may be recognized by cGAS, IFI16, or heterogeneous nuclear ribonucleoprotein A2B1 (hnRNPA2B1).^[^ ^8^, ^41^, ^42^ ^]^ ABCF1 has been uncovered as a DNA‐binding factor for the cytoplasmic double‐stranded DNAs involved in innate immune responses, including ISD, VACV‐70, and retroviral infection,^[^ ^16^ ^]^ but how ABCF1 recognizes DNA is unknown. Here, using both in vitro and in cells assay, we demonstrated ABCF1 as an HBV cccDNA‐binding protein to induce IFN production. Especially, the dCas9‐based CAPTURE assay verified the interaction of endogenous ABCF1 with native HBV‐derived cccDNA in HBV‐infected HepaRG^NTCP^ cells, while pull‐down assay detected the direct binding of recombinant ABCF1 proteins with cccDNA in vitro. In mechanism, ABCF1 interacts with cccDNA via a special Lys‐rich motif KKGKKDKKIKKT with four KK repeat, named KKx4. The positively charged amino acids, such as Arg and Lys, have been reported as important residues in DNA binding proteins, and are redundant in DNA protein binding regions,^[^ ^43^, ^44^ ^]^ suggesting that the ability of ABCF1 to interact with DNA may not be HBV and virus‐specific. Indeed, Retrovirus, similar to HBV, also produces episomal DNA circles and can be sensed by ABCF1.^[^ ^16^ ^]^ Besides, Choi E.B. et al. reported the binding of ABCF1 with short dsDNA that arises from genome instability in the nucleus.^[^ ^22^ ^]^ Furthermore, ABCF1 is also reported as a risk gene for autoimmune pancreatitis and arthritis.^[^ ^45^, ^46^ ^]^ Combined with our finding that ABCF1 binds cccDNA to enhance activation of innate immune signaling, all these evidences support ABCF1 as a dsDNA sensor that monitors pathogens infection and abnormal host DNA fragments.

Another important finding of this manuscript is that we link LLPS with cccDNA sensing and HBV transcription inhibition. Phase separation has emerged as a fundamental manner of cellular defense against viral infection and innate immune regulation.^[^ ^12^, ^47^ ^]^ Du et al. revealed that the cGAS “foci” are formed by the multivalent interactions between cGAS and DNA via LLPS, which facilitates the efficient production of cGAMP. Similarly, IFI16 initiates filament‐like LLPS after binding to HSV‐1 DNA to augment the induction of cytokines.^[^ ^14^ ^]^ Here, our data found that endogenous ABCF1 forms a foci‐like structure with cccDNA in HBV‐infected hepatocytes, and ABCF1‐cccDNA forms micrometer‐sized liquid droplets in vitro in different buffers. Importantly, the phase separation ability of ABCF1 is essential for its cccDNA binding and subsequent activation of innate immune responses and HBV transcription inhibition. Notably, ABCF1 compartmentalizes cccDNA from transcriptional machinery. In line with this, both in vitro binding assay and ChIP analysis showed that ABCF1 prevented Pol II and TBP loading to cccDNA in an LLPS‐dependent manner. Nevertheless, how ABCF1 activates TBK1 and IRF3 after sensing cccDNA is still unclear. A previous study suggested that ABCF1 interacts with multiple proteins including nucleic acid sensor HMGB2 and DNA sensor IFI16 to form a coordinated system for viral DNA detection.^[^ ^16^ ^]^ We speculated that ABCF1‐cccDNA condensates may form a molecular platform to recruit its partners or incorporate into the IFI16‐DNA phase to augment its activity, which needs further investigation.

Recently, ABCF1 has been implicated as a coactivator for transcription factor OCT4/SOX2.^[^ ^22^ ^]^ ABCF1‐LCD by itself is inactive in transcription and needs the assistance of the NBDs with known ATP hydrolysis in transcriptional coactivation.^[^ ^22^ ^]^ Different from its transcriptional activation in regulating pluripotency genes expression, we found ABCF1 executes a transcriptional inhibitory role in controlling cccDNA activity and this function was mediated by the LCD domain but not the NBD1 and NBD2 domains. ABCF1‐LCD is not a simple unstructured domain, it contains a polyQ tract that is essential for its phase separation with cccDNA. The presence of glutamine‐rich regions is also found in multiple P‐body components and contributes to P‐body assembly.^[^ ^48^ ^]^ Via polyQ‐mediated LLPS, ABCF1‐LCD might sequester cccDNA to induce its transcription silence. Similarly, the accumulation of aberrant short dsDNAs in damaged cells could bind ABCF1 and lead to the dissociation of ABCF1‐SOX2 interaction, which disrupts its role in activating stemness genes.^[^ ^22^ ^]^ In contrast, the polyQ tract acts as a flexible domain, allowing the flanking domains to come into close spatial proximity.^[^ ^49^ ^]^ Therefore, the flexible feature of ABCF1‐LCDs may facilitate the dynamic interaction with multiple partners to play different roles, either acting as a transcription activation or repression in a context‐dependent manner. Besides, similar to cccDNA, extrachromosomal circular DNA also exists in Kaposisarcoma‐associated herpesvirus (KSHV), Epstein–Barr virus (EBV), and human papillomavirus (HPV). Our findings imply that ABCF1 may serve as an important guardian in restricting the replication of multiple DNA viruses, which need further studies.

IFN‐I/III pathway activation is critical for HBV clearance, however, HBV is widely recognized as a “stealth” virus that does not invoke strong IFN responses.^[^ ^4^, ^50^ ^]^ Consistently, our data showed that HBV infection only induces weak expression of *IFNB1*, *IFNL1*, and *CXCL10*, which is augmented upon ectopic ABCF1 expression and attenuated upon ABCF1 knockdown. It has been reported that HBV circumvents endogenous innate immune recognition via multiple mechanisms to sustain persistent HBV infection. Expression of PRRs such as TLR3, RIG‐I, and MDA5 is downregulated in hepatocytes of CHB patients, which leads to the reduction of responsiveness of hepatocytes to HBV infection.^[^ ^51^ ^]^ Moreover, viral proteins (HBsAg, HBeAg, HBx) and HBV virion repress IFN‐β synthesis by targeting mitochondrial antiviral signaling (MAVS).^[^ ^5^ ^]^ HBx, as a multifunctional viral regulator, is essential for HBV cccDNA transcription and viral replication. Except for MAVS, there are multiple points of innate immune signaling pathways, including RIG‐I, TNF receptor‐associated factor 3 (TRAF3), cGAS, TBK1, IRF3, and adenosine deaminases acting on RNA 1 (ADAR1), can be targeted by HBx to negatively regulate the production of type I IFN.^[^ ^9^, ^50^ ^]^ Here, we found that HBx, but not other viral proteins, transcriptionally inhibited ABCF1 expression in a SOX4‐dependent manner, which may contribute to the evasion of ABCF1 recognition by HBV. All these literature and our study highlight the central role of HBx in bypassing innate immune recognition to maintain HBV replication.

In summary, our findings uncover the pivotal role of DNA sensor ABCF1 as a cccDNA interactor, which drives LLPS to activate IFN pathway and prevent Pol II loading from cccDNA, respectively. In turn, HBx transcriptionally represses ABCF1 expression to evade ABCF1‐mediated inhibition. This work broadens our understanding of the host‐HBV interaction and provides a novel strategy for HBV therapeutics.

## 4 Experimental Section

### Cell Culture

The human HCC cell lines Huh7, HepG2, HepG2^NTCP^, and Huh7^NTCP^ cells were maintained in Dulbecco's modified Eagle medium (DMEM, Gibco, Carlsbad, California, USA) supplemented with 10% fetal bovine serum (FBS) and 1% penicillin and streptomycin (PS). Human embryonic kidney cell line HEK293T was cultured in DMEM medium supplied with 10% FBS and 1% PS. HLCZ01 cell line ^[^ ^24^ ^]^ was gifted from Haizhen Zhu (Hunan University) and cultured with DMEM/F12 medium supplemented with 10% FBS, 1% PS, 40 ng mL^−1^ dexamethasone, and 10 ng mL^−1^ epidermal growth factor in collagen‐coated tissue culture plates. HepaRG^NTCP^ cells^[^ ^52^ ^]^ were kindly gifted from Yuchen Xia (Wuhan University) and maintained in Williams’ medium E (Gibco, Carlsbad, California, USA) supplemented with 10% FBS, 1% PS, 0.023 IE mL^−1^ insulin, 5 µg mL^−1^ hydrocortisone and 80 µg mL^−1^ gentamicin. The HepG2.2.15 cell line stably transformed with two copies of the HBV genome was maintained in MEM medium supplemented with 10% FBS, 200 µg mL^−1^ G418, and 2 mmol L^−1^ glutamine. Thirty‐eight HCC patient para‐tumor tissues were obtained from patients admitted to Qilu Hospital, Shandong University. The tissue specimens were snap‐frozen in liquid nitrogen and stored at −80 °C for RNA extraction. The use of resected human samples was approved by the Medical Ethical Committee of School of Basic Medical Sciences of Shandong University (Approval Number: ECSBMSSDU2022‐1‐70), and all participants were informed and provided written informed consent. A summary of the clinical information for the 38 patients is available in Table S1 (Supporting Information).

### Animal Experiments

Six‐week‐old male C57BL/6J mice purchased from Charles River Laboratories (Beijing, China) were fed under specific pathogen‐free conditions in the laboratory animal center of the School of Basic Medical Science of Shandong University. HBV carrier mice were prepared by hydrodynamic injection (HDI) of MC‐HBV plasmid (6 µg per mouse). To evaluate the role of ABCF1 in HBV replication, SB100 (0.8 µg per mouse), and ABCF1‐PT3EF1α (20 µg per mouse) plasmids were injected into mice at the same time as HDI. The total volume of plasmids was diluted in a volume of normal saline equivalent to 8% of the mouse's body weight and delivered into the tail veins within 5–8 s. All animal experimental procedures were performed in accordance with the Shandong University guidelines for animal experiments and approved by the Ethics Committee of the School of Basic Medical Sciences, Shandong University (Approval Number: ECSBMSSDU2022‐2‐117).

### Plasmids, Proteins, and siRNAs

Flag‐ABCF1 and Flag‐SOX4 expression plasmids were constructed by cloning their coding sequence into pCMV‐3×Flag 7.1 vector. The truncates of ABCF1 (LCD, ΔLCD, ΔNBD1, ΔNBD2, ΔLCD/NBD1, ΔLCD/NBD2) and the truncates of LCD (LCDΔpolyQ, LCDΔpolyE1, LCDΔpolyE2, LCDΔpolyQ‐FUS‐IDR, and LCDΔKKx4) were generated by KOD Kit (SMK‐101, TOYOBO, Japan). The pET‐22b‐mEGFP plasmid was kindly gifted by Professor Shuguo Sun (Huazhong University of Science and Technology). The prokaryotic expression plasmids of ABCF1 and truncates of ABCF1 (LCD, ΔLCD, LCDΔpolyQ, LCDΔpolyE1, LCDΔpolyE2, LCDΔpolyQ‐FUS‐IDR, and LCDΔKKx4) were constructed by cloning them into pET‐22b‐mEGFP vectors. The prokaryotic expression plasmids of TBP and truncates of ABCF1 (LCD and ΔLCD) were constructed by cloning them into pGEX‐6P‐1‐GST vectors. The recombinant proteins (GST‐LCD, GST‐ΔLCD, GST‐TBP) were purified by GST fusion protein purification kit (L0027, GenScript, USA). The recombinant proteins (GFP‐ABCF1, GFP‐LCD, GFP‐ΔLCD, GFP‐LCDΔpolyQ, GFP‐LCDΔpolyE1, GFP‐LCDΔpolyE2, GFP‐LCDΔpolyQ‐FUS‐IDR, GFP‐vector, GFP‐LCDΔKKx4, GFP‐KKx4, GFP‐polyQ, and GFP‐KKx4‐polyQ) were purified by Ni‐NTA beads (SA005100, Tiandirenhe, Jiangsu, China). The lenti‐CRISPR‐dCas9 (D10A/H840A) plasmid was generated by KOD Kit and plasmids (Biotin‐dCas9‐sgGal4, Biotin‐dCas9‐sgHBx and Biotin‐dCas9‐sgHBs) were constructed into lenti‐CRISPR‐dCas9 vector. HA‐HBx, HA‐HBc, HA‐HBsAg, and HA‐Pol expression plasmids were constructed by cloning their coding sequence into pCAGGS‐HA vector. All constructs were verified by sequencing. The minicircle‐HBV (MC‐HBV)^[^ ^23^ ^]^ and MC‐HBVΔHBx plasmids were constructed in the laboratory. The small interfering RNAs (siRNAs) specific for ABCF1 and SOX4 were synthesized from GenePharma (Shanghai, China) (Table S2, Supporting Information). The shABCF1 knockdown plasmids were constructed by cloning siABCF1 sequences into the pLVX‐puro‐GFP vector.

### Antibodies and Reagents

Antibodies for Western blot and ChIP assay: rabbit anti‐ABCF1 monoclonal antibody (HPA017578, Sigma, St. Louis, MO, USA), rabbit anti‐HBc polyclonal antibody (B0586, Dako, Copenhagen, Denmark), rabbit anti‐GST monoclonal antibody (#2625T, CST, Boston, MA, USA), rabbit anti‐IRF3 monoclonal antibody (#4302, CST), rabbit anti‐p‐IRF3 (#4947S, CST), rabbit anti‐TBK1 monoclonal antibody (#3504, CST), rabbit anti‐p‐TBK1 monoclonal antibody (#5483S, CST), rabbit anti‐SOX4 polyclonal antibody (27414‐1‐AP, Proteintech, Chicago, IL, USA), rabbit anti‐C/EBPα polyclonal antibody (#2295, CST), rabbit anti‐HNF1α polyclonal antibody (ab96777, abcam), mouse anti‐glyceraldehyde 3‐phosphate dehydrogenase (GAPDH) monoclonal antibody (60004‐1‐Ig, Proteintech), mouse anti‐GFP‐tag monoclonal antibody (66002‐1‐Ig, Proteintech), mouse anti‐Flag‐tag monoclonal antibody (M185‐3, MBL, Nagoya, Japan), mouse anti‐Cas9 monoclonal antibody (#14697T, CST), mouse anti‐Rpb1 CTD (4H8) monoclonal antibody (#2629, CST), and rabbit anti‐TBP antibody (#8515, CST).

The streptavidin dynabeads were purchased from Invitrogen (11205D, Thermo Fisher Scientific, Waltham, MA, USA). The anti‐GST magnetic beads were purchased from Beytime (P2138, Shanghai, China). The anti‐His magnetic beads were purchased from Beytime (P2135). The T5 exonuclease was purchased from Beytime (D7082S, Shanghai, China). The nucleic acid labeling kits (Biotin, MIR 3400, and Cy5, MIR 3725) were purchased from Mirus Bio LLC (Madison, WI, USA). The 1,6‐Hexanediol (240117) and Polyethylene glycol (PEG)‐8000 (89510) were purchased from Sigma (Sigma–Aldrich). The proteasome inhibitor MG132 (S1748) was purchased from Beytime (Shanghai, China) and the the protein synthesis inhibitor cycloheximide (CHX) (S7418) was purchased from Selleck (Shanghai, China). Chromatin immunoprecipitation Kits were purchased from Millipore (Merck KGaA, Darmstadt, Germany). The Coomassie Blue Super Fast Staining Solution was purchased from Beytime (P0017F). The Enhanced BCA Protein Assay Kit was purchased from Beytime (Shanghai, China). Plasmid‐safe DNase was purchased from Epicentre (Madison, WI, USA). In‐Fusion HD Cloning Kit was purchased from Beytime (D7010 M). Commercial enzyme‐linked immunosorbent assay kit for HBsAg and HBeAg was purchased from Dade Behring (Marburg, Germany).

### ChIP Assay

HepG2 cells were transfected with MC‐HBV and other plasmids, or HBV‐infected HLCZ01 cells were transfected with other plasmids for 72 h. The cells were fixed with 1% formaldehyde for 10 min at room temperature quenched by 0.125 mol L^−1^ glycine. The cell nucleus was isolated and sonicated at 25% amplitude, 10 s on, 10 s off, for 18 cycles to shear DNA. The protein‐DNA complexes were immunoprecipitated with indicated antibodies at 4 °C overnight, followed by immunoprecipitating with protein A/G magnetic beads at 4 °C for 1 h. Beads were then washed consecutively for 5 min on a rotating platform with 1 mL of each solution: Low salt wash buffer, High salt wash buffer, LiCl wash buffer, and TE wash buffer, followed by digesting with proteinase K and incubating for 2 h at 62 °C to release DNA fragments. The purified DNA was used for qPCR with specific primers.

### Protein Expression and Purification

All His‐tagged and GST‐tagged recombinant proteins were purified from *rosetta* (DE3) *E.coli* (CB108, TianGen, Beijing, China). Briefly, transformed appreciate DNA plasmid into *rosetta* (DE3) *E.coli* and picked a colony to culture with LB medium. Incubated at 37 °C until the OD_600_ is 0.6–0.8. Then the culture was induced to express proteins by adding 0.1 mm IPTG (ST098, Beytime) for 16–20 h at 16 °C. Lysis and sonication of the bacteria (Lysis buffer: 25 mm Tris‐HCl pH 7.5, 500 mm NaCl). Sonicated the suspension to shear the DNA until the turbidity was similar to that of a normal protein solution. The supernatant was purified by a gravity column and centrifuged by an ultrafilter.

### In Vitro Phase Separation Assay

MC‐HBV was labeled with Label IT Nucleic Acid Labeling kit Cy5 (MIR3725, Mirus) at 37 °C for 1 h and purified with a G50 microspin column according to the manufacturer's protocol. Phase separation of recombinant GFP‐proteins (mEGFP‐ABCF1, mEGFP‐LCD, mEGFP‐ΔLCD, mEGFP‐LCDΔpolyQ, mEGFP‐LCDΔpolyE1, mEGFP‐LCDΔpolyE2, mEGFP‐LCDΔpolyQ‐FUS‐IDR, mEGFP‐LCDΔKKx4, mEGFP‐KKx4, mEGFP‐polyQ, or mEGFP‐KKx4‐polyQ) with Cy5‐MC‐HBV were diluted into the indicated buffer with 10% PEG8000 for 5 min at 37 °C. The protein solution was added to a confocal glass plate and used LSM980 confocal microscope system (Zeiss) to observe images and the co‐localization was evaluated using the ImageJ software (NIH).

### Fluorescence Recovery after Photobleaching (FRAP)

FRAP experiments were performed on an LSM980 confocal microscope system (Zeiss). Fluorescent signals were bleached using the appropriate corresponding laser beam. The GFP‐ABCF1 and GFP‐LCD proteins were bleached using 488 nm lasers (30% intensity) and time‐lapse images were collected. The images were captured after the region of interest was photobleached. The experimental control was based on the quantification of fluorescence intensities of similar droplet regions without photobleaching. The FRAP results were analyzed by GraphPad Prism 7.0.

### MC‐HBV Biotinylation and Pull‐down

MC‐HBV was labeled with Label IT biotin reagent (MIR 3400, Mirus) at 37 °C for 1 h and purified with a G50 microspin column according to the manufacturer's protocol. Unlabeled MC‐HBV and 1 µg biotin‐MC‐HBV were incubated with 1 mg HepG2 cell lysate protein at 4 °C for overnight and then incubated for another 2 h with an extra 20 µL of Dynabeads M‐280 Streptavidin (11205D, Thermo Fisher Scientific). After careful washing with PBST 3 times, boiling with 25 µL 1× sodium dodecyl sulfate (SDS) loading buffer (P0015L, Beytime), and then protein samples were used for Western blot.

### DNA‐Pull Down and Streptavidin‐Pull‐Down Assays

The DNA‐pull down assay was performed as follows: MC‐HBV, GST‐tagged‐proteins or His‐tagged proteins, and anti‐GST magnetic beads or anti‐His magnetic beads were co‐added to protein‐DNA buffer (Glycerine, 1 m Tris‐HCl pH 8.0, 10 mg mL^−1^ BSA, 5 m NaCl, 5 m MgCl_2_, 0.5 m EDTA pH 8.0, 1 m DTT) for overnight at 4 °C. The beads were washed three times with PBST for 5 min each time, followed by digested with proteinase K in Elution buffer (1% SDS, 0.1 m NaHCO_3_, 5 m NaCl, 0.5 m EDTA, 1 m Tris‐HCl pH 6.5) and incubated for 2 h at 62 °C to release DNA fragments. The purified DNA was used for RT‐qPCR with cccDNA‐specific primers.

The streptavidin‐pull‐down assay was performed as follows: MC‐HBV plasmid, biotin‐labeled peptides (α‐helix1, α‐helix2, polyQ, and KKx4 of LCD), and streptavidin magnetic beads were co‐added to PBS buffer for overnight at 4 °C. The beads were washed three times with PBST for 5 min each time, followed by digesting with proteinase K in Elution buffer and incubating for 2 h at 62 °C to release DNA fragments, and their binding with MC‐HBV was analyzed by streptavidin‐pull‐down assay. The purified DNA was used for RT‐qPCR with cccDNA‐specific primers.

### Dual Luciferase Reporter Assay

The Huh7 cells were transfected into indicated plasmids. Approximately 48 hours after transfection, the cells were lysed and subjected to luciferase activity analysis using a Dual‐luciferase reporter assay system (Promega, Madison, USA).

### Establishment of Genomic‐Tagged Cell Line

CRISPR‐Cas9 was used for genome editing to generate N‐terminal tagged mEGFP‐ABCF1‐HepG2^NTCP^ cell lines. The guide RNA targeting the N terminus of ABCF1, which spans the start codon region of ABCF1, was cloned into the Lenti‐CRISPR‐Cas9 vector (Cas9‐ABCF1‐gRNA). Repair templates were cloned into a pUC19 vector (pUC19‐mEGFP), containing the mEGFP, and two homology arms flanking the insert. HepG2^NTCP^ cells were transfected with 5 µg Cas9‐ABCF1‐gRNA plasmid and 4 µg pUC19‐mEGFP plasmid. Seven days post‐transfection, mEGFP‐positive cells were sorted into 96‐well plates for culture, and Western blot assay was performed to determine whether mEGFP was successfully knocked in.

### Preparation, Infection, and Detection of EdU‐HBV

HepG2.2.15 cells were cultured in MEM medium supplemented with 10 µm EdU, 2% FBS, 200 µg mL^−1^ G418, and 2 mmol L^−1^ glutamine. The supernatant was collected every two days and filtered with a 0.45 µm filter to discard the cell debris. Then 6% PEG8000 was added at the final concentration and rotated slowly for 4–6 h at 4 °C. EdU labeled HBV (EdU‐HBV) was concentrated through centrifuging 20 min at 4000 rpm, resuspended with MEM medium and titered the HBV DNA copies. The mEGFP‐ABCF1‐knockin HepG2^NTCP^ cells were infected with EdU‐HBV at indicated Geq and fixed with 4% paraformaldehyde, then permeabilized with 0.5% Triton X‐100 in PBS and labeled the EdU molecules through click chemistry with Click‐iT Plus EdU Alexa Fluor 647 Imaging Kit (C10640, Thermo Fisher Scientific) for 30 min at room temperature. After staining with DAPI, Alexa‐647 positive HBV cccDNA in the nucleus was imaged using a laser scanning confocal microscope. Co‐localization was evaluated using the Coloc 2 plugin of image ImageJ software (NIH) to calculate Pearson's coefficients of EdU‐cccDNA co‐localization with ABCF1.

### Enzyme‐Linked Immunosorbent Assay

HBsAg and HBeAg secreted into cell culture supernatant were measured using commercially available HBsAg or HBeAg Enzyme‐Linked Immunosorbent Assay kits (InTec, Inc, Xiamen, China) as protocol. The antigen levels were quantitated at OD_450/630_ optical density and represented the relative levels to scramble.

### HBV Infection in Cultured Cells

Cell‐culture‐derived HBV stock was prepared from HepG2.215 culture supernatant. Briefly, HBV stocks were concentrated from the supernatants of HepG2.215 cells using a centrifugal filter (Millipore) and titrated using a Hepatitis B Viral DNA quantitative fluorescence diagnostic kit (Sansure Biotech, Changsha, China). Aliquoted HBV stocks were stored at −80 °C.

For infection, HepG2^NTCP^ or HepaRG^NTCP^ cells were infected with 400 genomes per cell of HBV in a medium containing 4% polyethylene glycol 8000 (Sigma‐Aldrich, USA) for 12 h, and then washed the cells three times with phosphate‐buffered saline (PBS) and cultured in the DMEM with 2% FBS for 4–7 days, followed by indicated experiments.

### CHX Chase Assay

Huh7 cells were transfected with Flag‐SOX4 or HA‐HBx plasmids for 36 h, the medium was then replaced with medium containing 500 µg mL^−1^ CHX, and the cells were further incubated at 37 °C for the indicated time points from 0 to 120 min. Changes in SOX4 protein levels were analyzed by Western blot. The SOX4 protein half‐life was determined using GraphPad Prism 7.0.

### Transfection and RT‐qPCR

The cells were grown overnight and the cell density reached 70%‐80%, then transfected with plasmids using transfection reagents Lipofectamine 2000 (11668019, Thermo Fisher Scientific) according to the manufacturer's protocol, after 6 h of transfection, changed the cell medium. Total RNA was extracted from cells using TRIzol reagent (DP424, TianGen), and 1 µg RNA was used to synthesize complementary DNA using a PrimeScript RT Reagent Kit with genomic DNA Eraser (2641A, Takara, Kyoto, Japan). RT‐qPCR analysis of gene expression was performed using SYBR mix (FP205‐02, Vazyme, Nanjing, China) according to the manufacturer's protocol by the indicated primers (Table S2, Supporting Information). Glyceraldehyde‐3‐phosphate dehydrogenase (GAPDH) was used as an internal control. Relative gene expression was normalized to GAPDH using the 2^–ΔΔCt^ method.

### Statistical Analysis

All statistical analyses were performed using GraphPad Prism 7.0 software. Unpaired Student's *t*‐test and one‐way ANOVA were performed to determine the statistical significance of differences between groups. Significance levels are indicated by asterisks: *p < 0.05; **p < 0.01; NS.: non‐significant.

## Conflict of Interest

The authors declare no conflict of interest.

## Author Contributions

C.M. and Z.W. conceptualized and supervised the study; C.M. and Z.W. provided funding; C.R. performed all experiments; Z.Z. and Y.D. helped to construct plasmids; Z.F., L.W., and Y.S. helped to perform HBV infection assays; Y.D. and K.W. assisted with animal experiments and RT‐qPCR; S.S., Y.F., X.Y., C.L., C.G., L.G., and X.L. contributed to the design of the project and extensive discussions; Z.W. and C.M. wrote the manuscript and the other authors revised it.

## Supporting information



Supporting Information

## Data Availability

The data that support the findings of this study are available from the corresponding author upon reasonable request.
