# Supplementary material for: DNA Sensor ABCF1 Phase Separates With cccDNA to Inhibit Hepatitis B Virus Replication
Source: Adv Sci (Weinh). 2024 Nov 5;11(48):2409485. doi: 10.1002/advs.202409485 (PMC11672287; doi:10.1002/advs.202409485)
Supplement: Supplementary file 1 — Supporting Information [file ADVS-11-2409485-s001.docx]

Supporting Information

**DNA Sensor ABCF1 Phase Separates with cccDNA to Inhibit Hepatitis B Virus Replication**

*Caiyue Ren, Zhaoying Zhang, Yutong Dou, Yang Sun, Zhendong Fu, Liyuan Wang, Kai Wang, Chengjiang Gao, Yuchen Fan, Shuguo Sun, Xuetian Yue, Chunyang Li, Lifen Gao, Xiaohong Liang, Chunhong Ma* and Zhuanchang Wu**

Dr. C. Ren, Dr. Z. Zhang, Dr. Y. Dou, Dr. Y. Sun, Dr. Z. Fu, Dr. L. Wang, Dr. K. Wang, Prof. C. Gao, Prof. L. Gao, Prof. X. Liang, Prof. C. Ma, Prof. Z. Wu

Key Laboratory for Experimental Teratology of Ministry of Education and Department of Immunology, School of Basic Medical Sciences, Cheeloo Medical College, Shandong University, Jinan, 250012, Shandong, China

Email: [zhuanchangwu@sdu.edu.cn](mailto:zhuanchangwu@sdu.edu.cn)

Email: [machunhong@sdu.edu.cn](mailto:machunhong@sdu.edu.cn)

Prof. Y. Fan

Department of Hepatology, Qilu Hospital, Cheeloo Medical College, Shandong University, 250012 Jinan

Prof. S. Sun

Department of Human Anatomy, Histology and Embryology, School of Basic Medicine, Tongji Medical College, Huazhong University of Science and Technology, 430030 Wuhan, Hubei, China

Prof. X. Yue

Department of Cellular Biology, School of Basic Medical Sciences, Shandong University, 250012 Jinan, China

Prof. C. Li

Key Laboratory for Experimental Teratology of the Ministry of Education, Department of Histology and Embryology, School of Basic Medical Sciences, Shandong University, 250012 Jinan, China

**
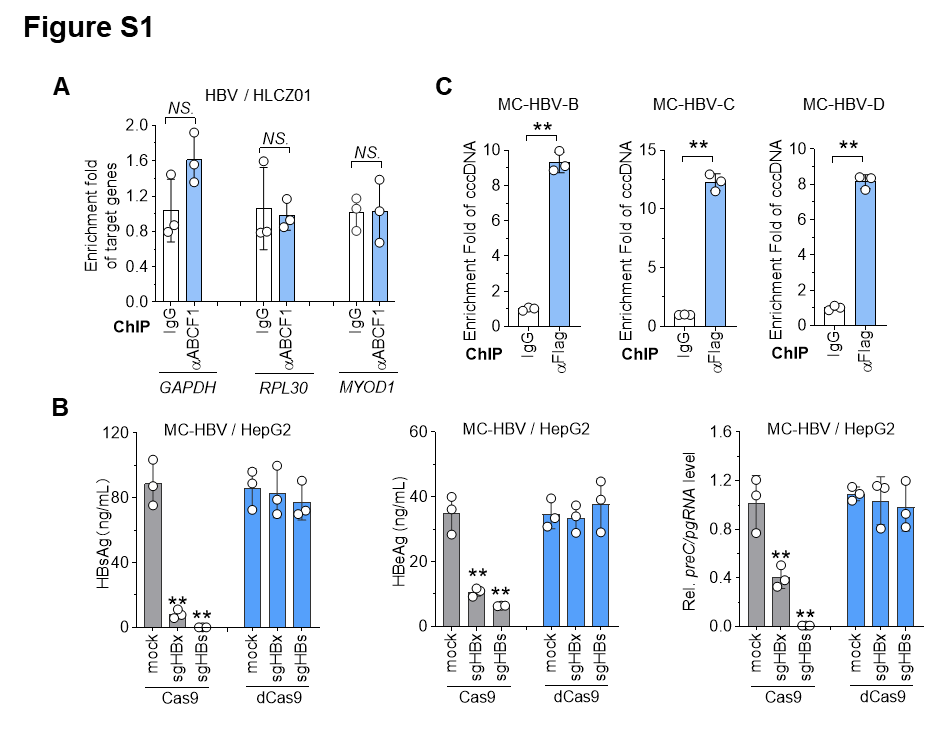
**

**Figure S1. ABCF1 senses HBV cccDNA from different genotypes.** (A) ChIP analysis of ABCF1-gDNA interaction. The enrichment of ABCF1 on ***GAPDH*, *RPL30*, and *MYOD1*** genes was measured by ChIP-qPCR. n = 3 biologically independent samples. (B) dCas9-sgRNA did not affect HBV replication. Cas9-sgHBV or dCas9-sgHBV were cotransfected with MC-HBV into HepG2 cells for 3 days, the levels of HBsAg and HBeAg were measured by ELISA and the levels of *preC/pgRNA* were detected by RT-qPCR. n = 3 biologically independent samples. (C) ChIP analysis of the interaction of ABCF1 with HBV cccDNA from different genotypes. Genotype B, C, or D MC-HBV were co-transfected with ABCF1 plasmids into HepG2 cells for 3 days, respectively, and the enrichment of ABCF1 on cccDNA was measured by ChIP-qPCR with cccDNA-specific primers. n = 3 biologically independent samples. Results are representative of three independent experiments. *P* values were determined by unpaired two-tailed t-tests (A-C); ***P* < 0.01, *NS.*: non-significant.

**
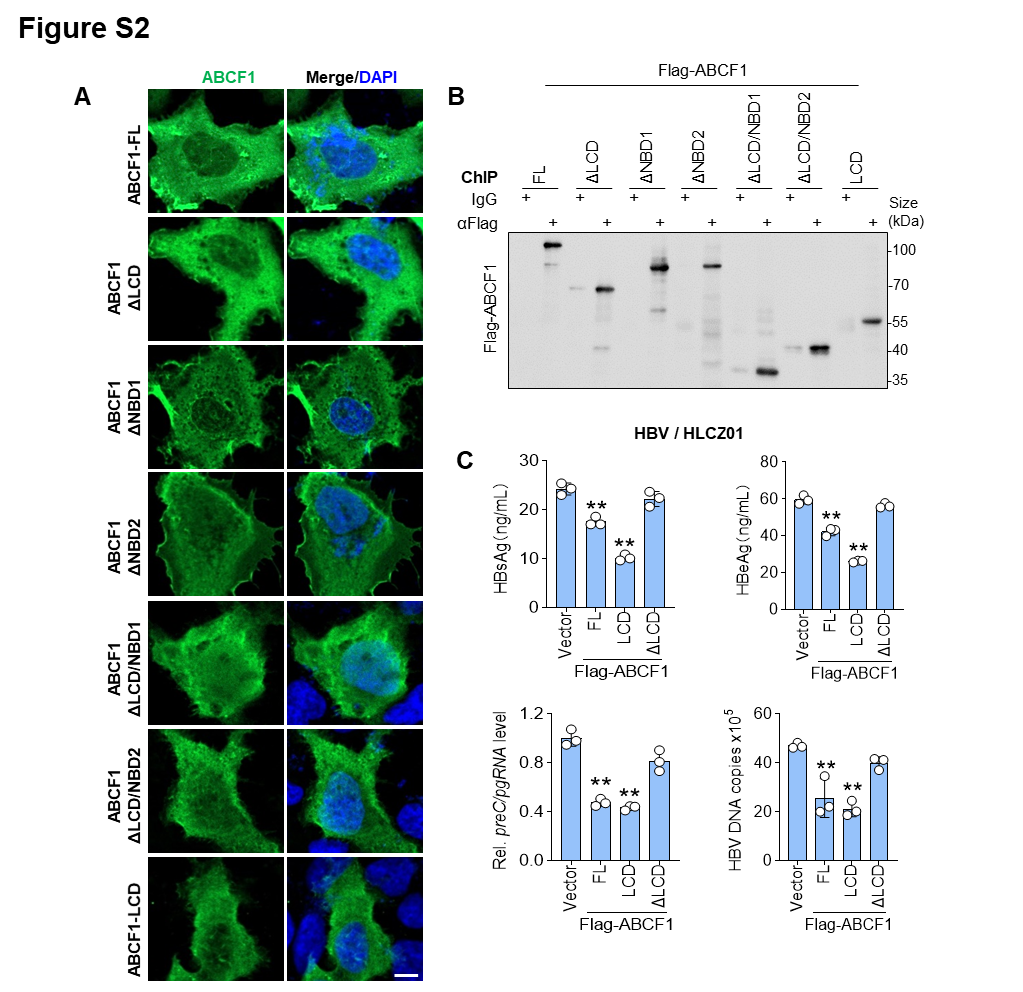
**

**Figure S2.** **LCD domain is required for ABCF1 to bind cccDNA and suppress HBV replication.** (A) The subcellular localization of different ABCF1 truncates. A series of ABCF1 truncates were transfected into Huh7 cells for 48 h and their subcellular localization was analyzed by Immunofluorescence staining. Scale bar, 10 μm. (B) Western blots showing the efficiency of immunoprecipitation in HepG2 cells. A series of ABCF1 truncates were cotransfected with MC-HBV into HepG2 cells for 72 h, and ChIP assays were performed with anti-Flag-ABCF1. The efficiency of immunoprecipitation was evaluated by Western blot. (C) ABCF1 inhibits HBV transcription through LCD domain in HBV-infected HLCZ01 cells. ABCF1 constructs were transfected into HBV-infected HLCZ01 cells for 3 days, and viral HBsAg, HBeAg (ELISA), *preC/pgRNA* (RT-qPCR), and HBV DNA (qPCR) levels were measured. n = 3 biologically independent samples. Results are representative of three independent experiments. *P* values were determined by unpaired two-tailed t-tests (C); ***P* < 0.01.

**
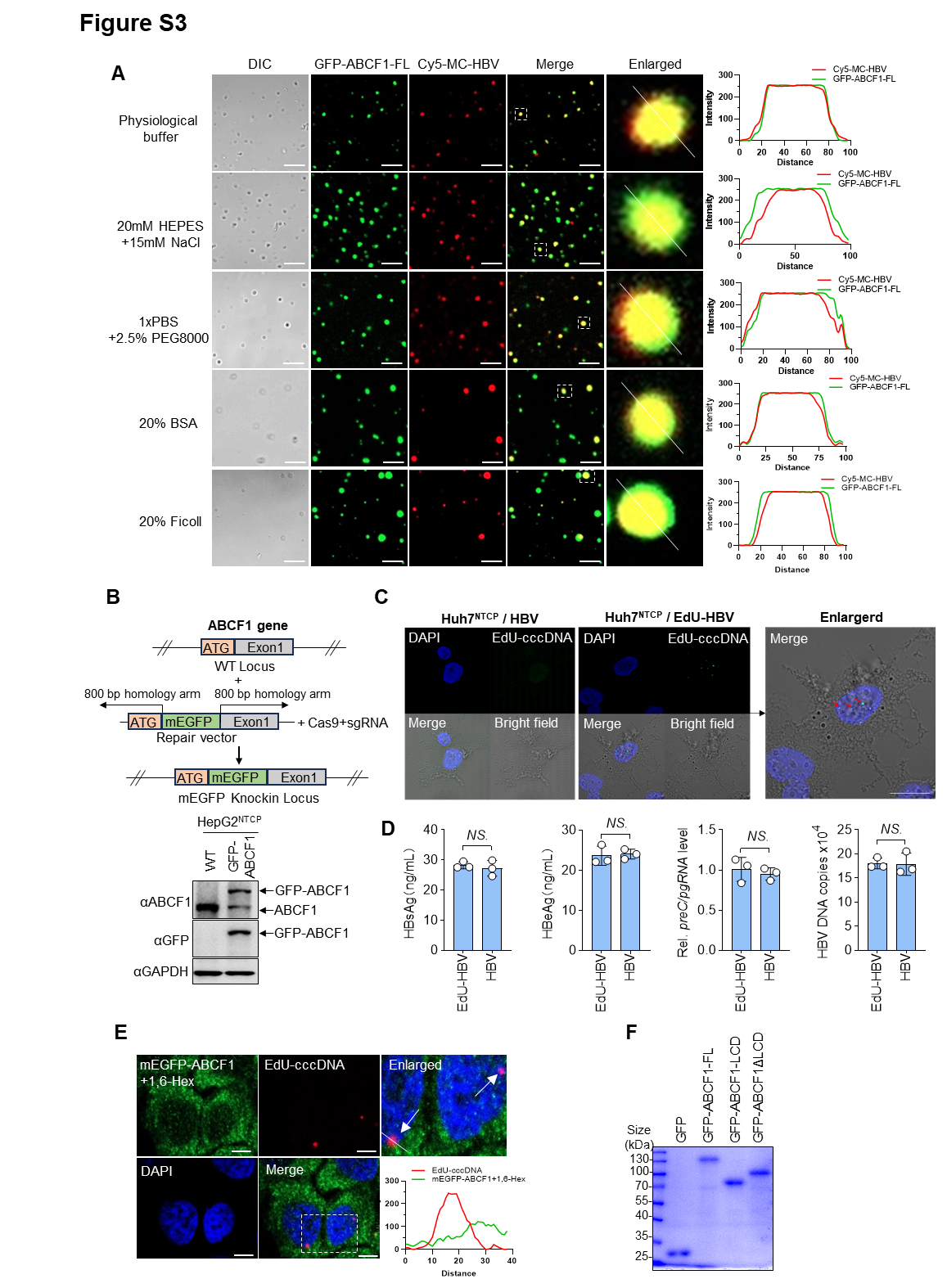
**

**Figure S3. LCD of ABCF1 interacts with cccDNA to form phase-separated condensates.** (A) Phase separation of ABCF1 and cccDNA in different buffers. Recombinant GFP-ABCF1 was incubated with Cy5-MC-HBV for 5 min at 37℃ in physiological buffer, NaCl buffer, 2.5% PEG8000 buffer, 20% BSA buffer, or 20% Ficoll buffer, and the formation of liquid droplets was imaged. The intensity profiles correspond to the white lines drawn in the enlarged images. Scale bar, 10 μm. (B) Construct of mEGFP-ABCF1 knockin HepG2^NTCP^ cell lines. Knockin locus (upper panel) and Western blot detection (lower panel) of mEGFP-ABCF1. (C) EdU-HBV HBV virion was metabolically labeled with EdU in HepG2.2.15 cells and infected Huh7^NTCP^ cells for 12 h. Cells were fixed to stain HBV cccDNA with Alexa Fluor 488 dye through Click-iT method and observed with laser scanning confocal microscope, arrow label represented HBV cccDNA. Scale bars, 10 μm. (D) Analysis of EdU-HBV and HBV replication levels. HepaRG^NTCP^ cells were infected with EdU-HBV or HBV at 400 Geq for 5 days. HBsAg and HBeAg levels in cell culture supernatant were detected by ELISA, intracellular *preC/pgRNA* and supernatant HBV DNA were analyzed by RT-qPCR and qPCR, respectively. n = 3 biologically independent samples. (E) Effect of 1,6-Hex on ABCF1-cccDNA puncta formation in live HBV-infected HepG2^NTCP^ cells. EdU-HBV virion-infected mEGFP-ABCF1 knockin HepG2^NTCP^ cells for 72 h, followed by 2.5% 1,6-Hex treatment for 2 h, and ABCF1-cccDNA puncta were analyzed by confocal microscopy. Scale bars, 10 μm. (F) The recombinant GFP-ABCF1 proteins (ABCF1, LCD, and ΔLCD) were purified and detected by Coomassie staining. Results are representative of three independent experiments. *P* values were determined by unpaired two-tailed t-tests (D); *NS.*: non-significant.

**
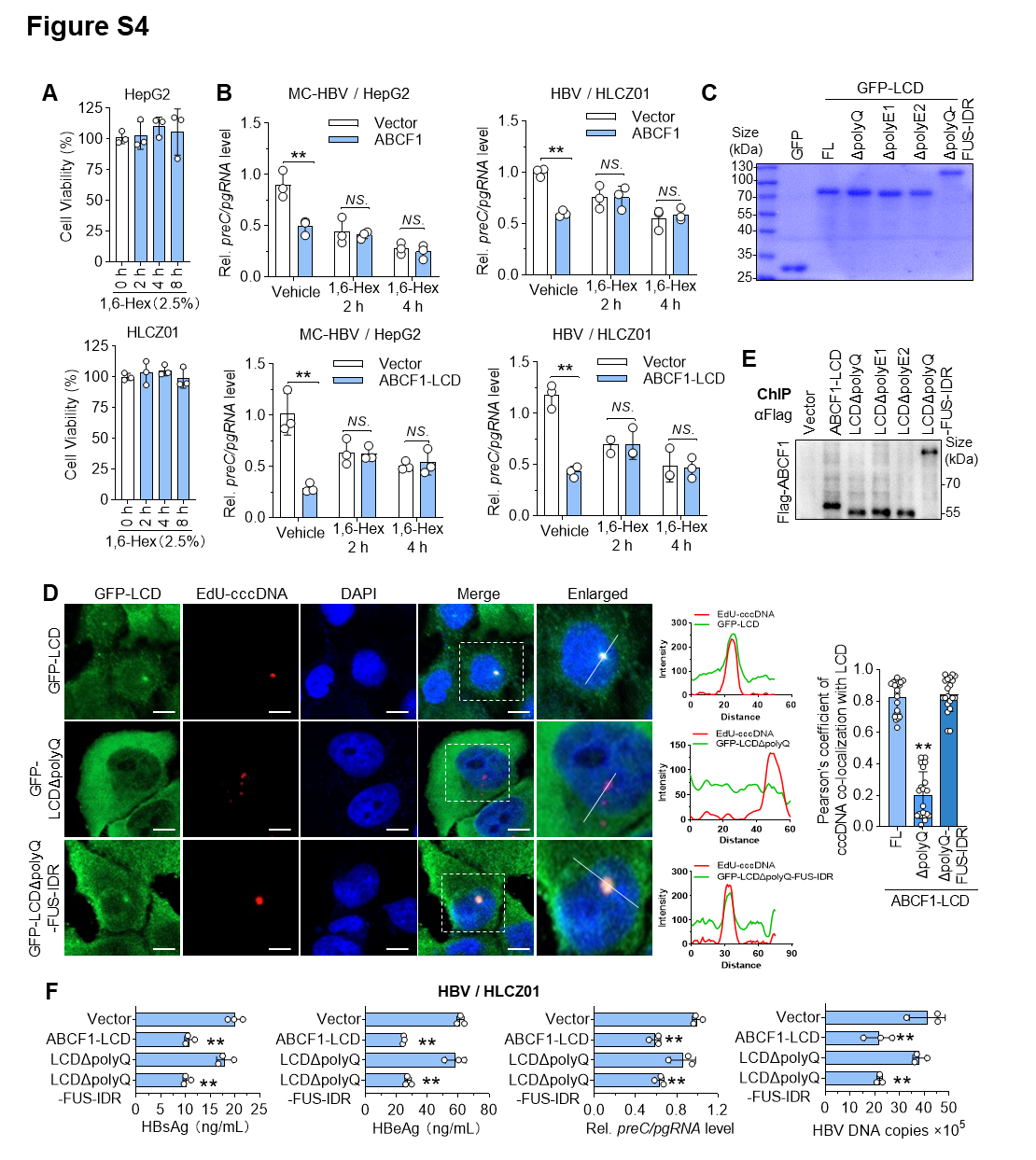
**

**Figure S4. ABCF1 inhibits HBV replication through LLPS.** (A) Cytotoxicity of 1,6-Hex in hepatocytes. HepG2 cells and HLCZ01 cells were treated with 2.5% 1,6-Hex at different times, and the cell viability was measured by CCK-8 assay. n = 3 biologically independent samples. (B) Disruption of ABCF1-mediated transcriptional repression on cccDNA by 1,6-Hex *in vivo*. ABCF1 and ABCF1-LCD were overexpressed in MC-HBV-transfected HepG2 cells and HBV-infected HLCZ01 cells for 48 h, followed by 2.5% 1,6-Hex treatment for 2 h and 4 h, and HBV *preC/pgRNA* levels were quantified by RT-qPCR. n = 3 biologically independent samples. (C) The recombinant GFP-ABCF1 proteins (LCD, ΔpolyQ, ΔpolyE1, ΔpolyE2, and ΔpolyQ-FUS-IDR) were purified and detected by Coomassie staining. (D) Immunofluorescence assay of LCD-cccDNA puncta formation. HepG2^NTCP^ cells were infected with EdU-labeled HBV at 400 Geq for 24 h and then transfected with GFP-ABCF1-LCD, GFP-LCDΔpolyQ, or GFP-LCDΔpolyQ-FUS-IDR plasmids for 2 days. The puncta were imaged by confocal microscopy and their colocalization was analyzed by Pearson’s coefficient. Scale bars, 10 μm. (E) The immunoprecipitation efficiency of ChIP assay in Figure 5D was evaluated by Western blot. (F) Phase separation of ABCF1-cccDNA determines the inhibitory role of ABCF1 on HBV replication in HBV-infected HLCZ01 cells. LCD constructs were transfected into HBV-infected HLCZ01 cells for 3 days, and viral HBsAg, HBeAg (ELISA), *preC/pgRNA* (RT-qPCR), and HBV DNA (qPCR) levels were measured. n = 3 biologically independent samples. Results are representative of three independent experiments. *P* values were determined by unpaired two-tailed t-tests (B, D, and F); ***P* < 0.01, *NS.*: non-significant.


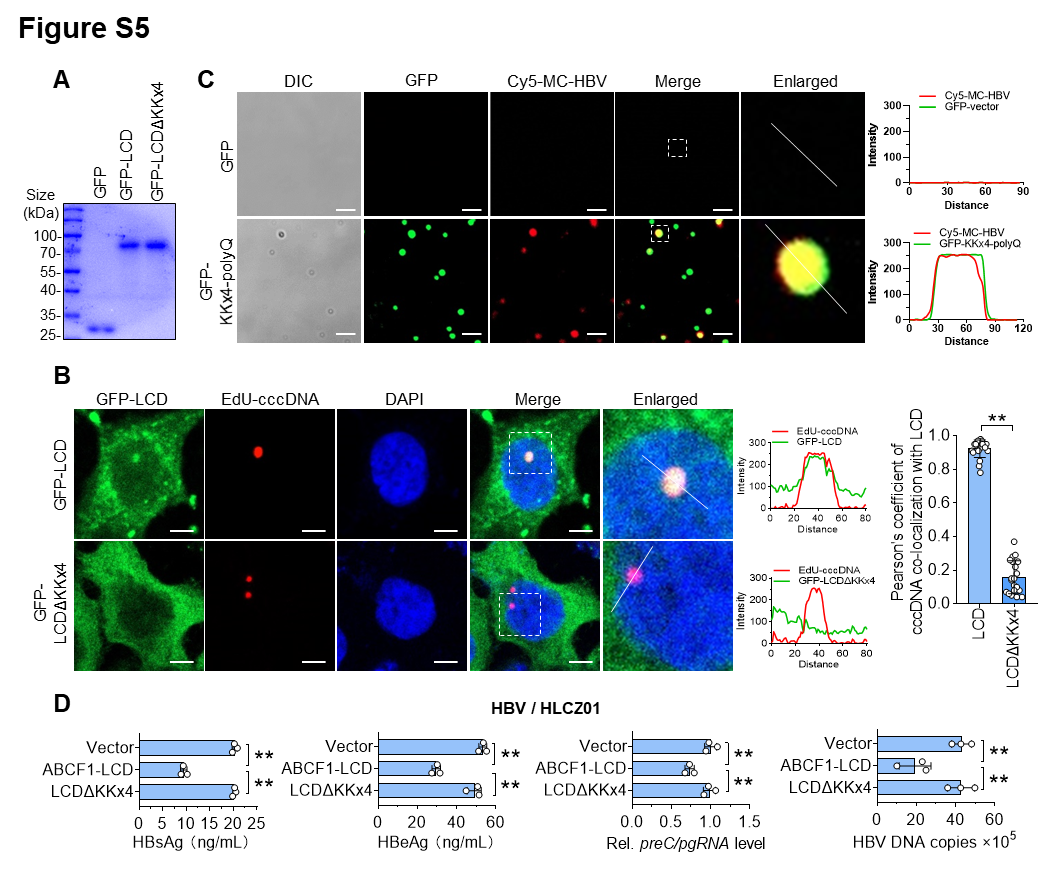


**Figure S5. KKx4 motif dominates LLPS of ABCF1-LCD and cccDNA and ABCF1-mediated HBV suppression.** (A) The recombinant GFP-ABCF1-LCD and GFP-ABCF1-LCDΔKKx4 proteins were purified and detected by Coomassie staining. (B) Immunostaining of LCD-cccDNA puncta formation in HepG2^NTCP^ cells. HepG2^NTCP^ cells were infected with EdU-labeled HBV at 400 Geq for 24 h and then transfected with GFP-LCD, or GFP-LCDΔKKx4 plasmids for 2 days. The puncta were imaged by confocal microscopy. The localization of GFP-LCD or GFP-LCDΔKKx4 with EdU-cccDNA was analyzed by calculating Pearson’s coefficient (right panel). Scale bars, 10 μm. (C) Phase separation of GFP-KKx4-polyQ with cccDNA. Recombinant GFP-KKx4-polyQ protein was incubated with Cy5-MC-HBV for 5 min at 37℃ in 10% PEG8000 buffer and the formation of liquid droplets was imaged. The intensity profiles correspond to the white lines drawn in the enlarged images. Scale bar, 10 μm. (D) KKx4 is critical for ABCF1-mediated HBV inhibition. ABCF1-LCD or LCDΔKKx4 was transfected into HBV-infected HLCZ01 cells for 3 days, and levels of HBsAg, HBeAg, *preC/pgRNA*, and HBV DNA were measured by ELISA, RT-qPCR, and qPCR, respectively. n = 3 biologically independent samples. Results are representative of three independent experiments. *P* values were determined by unpaired two-tailed t-tests (B, D); ***P* < 0.01.

**
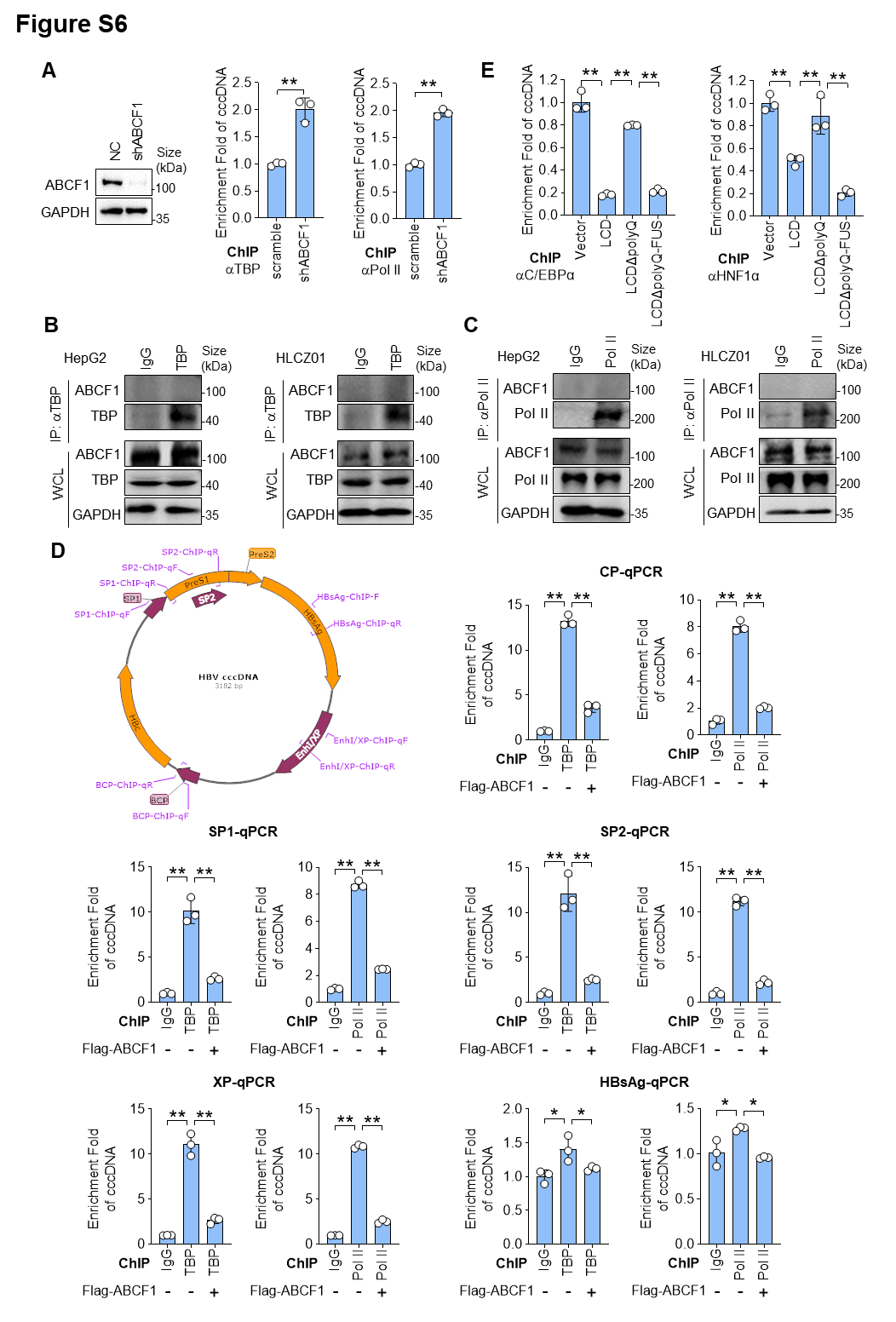
**

**Figure S6. ABCF1 inhibits the occupancy of Pol Ⅱ on cccDNA promoters.** (A) ABCF1 knockdown promotes the recruitment of TBP and Pol Ⅱ to cccDNA. ABCF1-knockdown HepG2 cells were transfected with MC-HBV for 3 days, the enrichment of TBP and Pol Ⅱ on cccDNA was analyzed by ChIP assay using either anti-TBP or anti-Pol Ⅱ (scramble siRNA transfected cells as control), and the knockdown efficiency of ABCF1 was evaluated by Western blot. n = 3 biologically independent samples. (B, C) Co-IP was performed with an anti-TBP antibody (B) or anti-Pol Ⅱ antibody (C) in HepG2 and HLCZ01 cells to evaluate the interaction of ABCF1 with RNA Pol Ⅱ complex. Results are representative of two independent experiments. (D) ABCF1 represses the binding of TBP and Pol Ⅱ to HBV promoters. ABCF1 was transfected into MC-HBV-transfected HepG2 cells for 3 days, the enrichment of TBP and Pol Ⅱ on cccDNA was measured by ChIP using either anti-TBP or anti-Pol II, IgG as control. qPCR was included to amplify the binding sequence using HBV promoter-specific primers (CP, SP1, SP2, XP) and HBsAg-coding gene primers. The primers were indicated in purple in HBV cccDNA map shown in the up-left panel. n = 3 biologically independent samples. (E) Phase separation is required for ABCF1-mediated inhibition of C/EBPα and HNF1α occupancy on cccDNA. ABCF1-LCD, LCDΔpolyQ, and LCDΔpolyQ-FUS-IDR were overexpressed in MC-HBV-transfected HepG2 cell models for 3 days, the enrichment of C/EBPα and HNF1α on cccDNA was analyzed by ChIP assay, n = 3 biologically independent samples. Results are representative of two independent experiments. *P* values were determined by unpaired two-tailed t-tests (A, D, and E); **P* < 0.05, ***P* < 0.01.

**
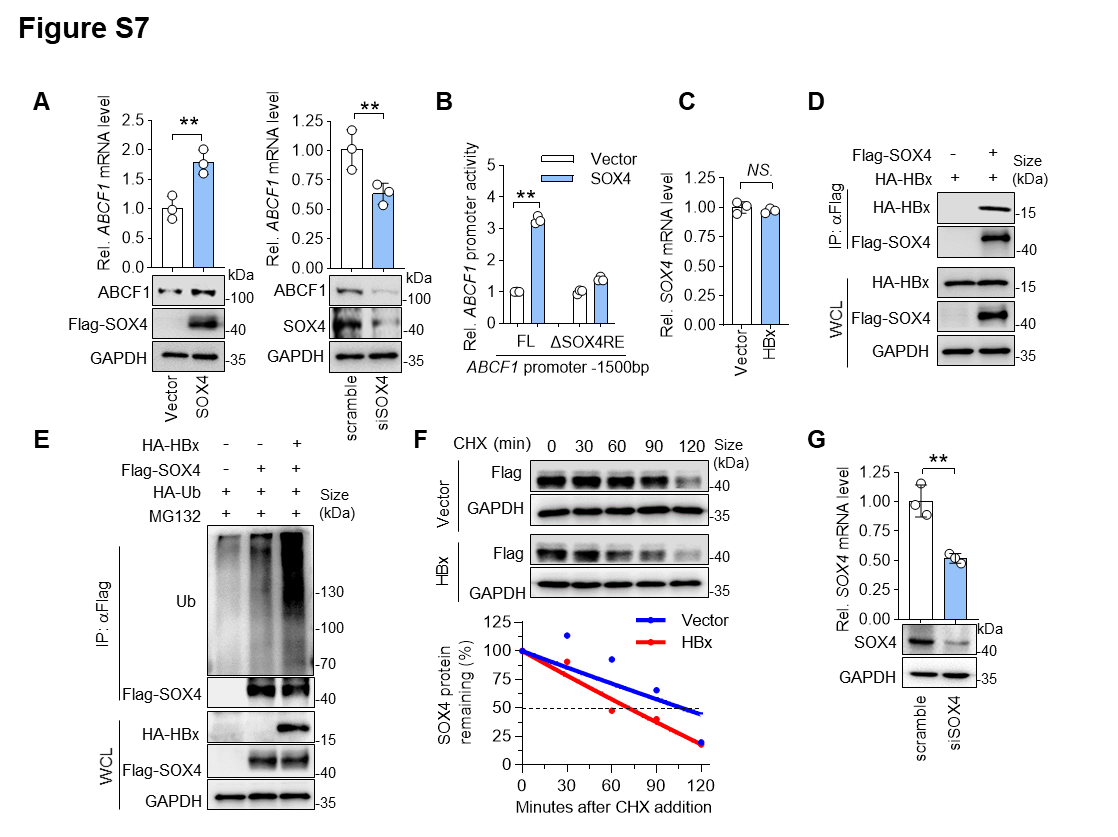
**

**Figure S7.** **HBx transcriptionally inhibits ABCF1 expression.** (A) Analysis of ABCF1 mRNA and protein levels upon SOX4 overexpression or knockdown. Flag-SOX4 plasmid or SOX4-siRNA was respectively transfected into Huh7 cells for 72 hours, and the mRNA and protein levels of ABCF1 were detected by RT-qPCR and Western blot, respectively. n = 3 biologically independent samples. (B) Measure of ABCF1 promoter activity upon SOX4 overexpression. Huh7 cells were cotransfected with Flag-SOX4 plasmid and ABCF1 promoter-reporter or mutant ABCF1 promoter-reporter with deletion of SOX4 binding motif (ΔSOX4RE) for 48 hours, the dual-luciferase assay was performed to detect ABCF1 promoter activity. n = 3 biologically independent samples. (C) HBx did not affect SOX4 transcription. HA-HBx plasmid was transfected into Huh7 cells for 48 hours, and the mRNA level of SOX4 was detected by RT-qPCR. n = 3 biologically independent samples. (D) The interaction between HBx and SOX4. HA-HBx and Flag-SOX4 plasmids were co-transfected into HEK293T cells for 48 h. Co-IP was performed with an anti-Flag antibody to analyze the interaction of HBx with SOX4. (E) HBx promotes the ubiquitination of SOX4. Flag-SOX4 was cotransfected with HBx and HA-Ub into HEK293T cells for 48 hours, and then treated with MG132 for 6 hours. Flag-SOX4 was immunoprecipitated and immunoblotted with anti-Ub antibody to measure its ubiquitylation. (F) CHX chase assay of SOX4 half-life after HBx overexpression. Huh7 cells were transfected with Flag-SOX4 and HA-HBx for 36 h. Cells were then treated with CHX at 500 μg/mL. SOX4 protein levels at indicated time points were detected by Western blot. Relative SOX4 protein levels normalized to GAPDH were presented relative to the level (set as 100%) at 0 h post-CHX treatment. (G) The knockdown efficiency of SOX4. SOX4-siRNA was transfected into Huh7 cells for 72 hours, the mRNA and protein levels of SOX4 were detected by RT-qPCR and Western blot respectively, GAPDH as internal control. n = 3 biologically independent samples. Results are representative of three independent experiments. *P* values were determined by unpaired two-tailed t-tests (A, B, C, and G); ***P* < 0.01, *NS.*: non-significant.

**Supporting Tables**

**Table S1 Clinical characteristics of enrolled subjects**

| **Characteristics** | **No. of patients** |  |
| --- | --- | --- |
| **Age (year)**  ≤55  ＞55 | 16  22 |  |
| **Gender**  Male  Female  **HBsAg**  Positive  Negative  **Differentiation**  I-II  III-IV | 30  8  32  6  28  10 |  |

**Table S2 siRNA and primers in this study**

| **Gene** | **Forward primer (5’-3’)** | **Reverse primer (5’-3’)** |
| --- | --- | --- |
| *ABCF1* | GGCTTCTTGGATGATGTCTGCAC | GCCTTCAGCTCCTTCAGCTTTTT |
| cccDNA | TTCTCATCTGCCGGACCG | CACAGCTTGGAGGCTTGAAC |
| *CXCL10* | GGTGAGAAGAGATGTCTGAATCC | GTCCATCCTTGGAAGCACTGCA |
| *GAPDH* | GGAGTCCACTGGCGTCTTCAC | GAGGCATTGCTGATGATCTTGAGG |
| *IFNB1* | CTTGGATTCCTACAAAGAAGCAGC | TCCTCCTTCTGGAACTGCTGCA |
| *IFNL1* | AACTGGGAAGGGCTGCCACATT | GGAAGACAGGAGAGCTGCAACT |
| *preC/pgRNA* | CTCAATCTCGGGAATCTCAATGT | AGGATAGAACCTAGCAGGCATAAT |
| *RPL30*-ChIP  *GAPDH*-ChIP  *MYOD1*-ChIP | CAAGGCAAAGCGAAATTGGT  TCGACAGTCAGCCGCATCT  CCGCCTGAGCAAAGTAAATGA | GCCCGTTCAGTCTCTTCGATT  CTAGCCTCCCGGGTTTCTCT  GGCAACCGCTGGTTTGG |
| **siRNA** | **siRNA sequence (5’-3’)** |  |
| siABCF1-1 | GCAAGGAGCUGUUCGUCAATT |  |
| siABCF1-2  siSOX4-1  siSOX4-2 | GCUGUCAUCUGGCUUAAUATT  AUCUGAAUUGGUACUGGAUAAU  UCGAGCUUCCCCTAUCAUGG |  |
